# Navigational Behavior of Humans and Deep Reinforcement Learning Agents

**DOI:** 10.3389/fpsyg.2021.725932

**Published:** 2021-09-22

**Authors:** Lillian M. Rigoli, Gaurav Patil, Hamish F. Stening, Rachel W. Kallen, Michael J. Richardson

**Affiliations:** ^1^School of Psychological Sciences, Macquarie University, Sydney, NSW, Australia; ^2^Centre for Elite Performance, Expertise and Training, Macquarie University, Sydney, NSW, Australia

**Keywords:** task-dynamical model, dynamical perceptual motor primitives, deep reinforcement learning, navigational behavior, obstacle avoidance, route selection

## Abstract

Rapid advances in the field of Deep Reinforcement Learning (DRL) over the past several years have led to artificial agents (AAs) capable of producing behavior that meets or exceeds human-level performance in a wide variety of tasks. However, research on DRL frequently lacks adequate discussion of the low-level dynamics of the behavior itself and instead focuses on meta-level or global-level performance metrics. In doing so, the current literature lacks perspective on the qualitative nature of AA behavior, leaving questions regarding the spatiotemporal patterning of their behavior largely unanswered. The current study explored the degree to which the navigation and route selection trajectories of DRL agents (i.e., AAs trained using DRL) through simple obstacle ridden virtual environments were equivalent (and/or different) from those produced by human agents. The second and related aim was to determine whether a task-dynamical model of human route navigation could not only be used to capture both human and DRL navigational behavior, but also to help identify whether any observed differences in the navigational trajectories of humans and DRL agents were a function of differences in the dynamical environmental couplings.

## Introduction

Rapid advances in the field of Deep Reinforcement Learning (DRL; Berner et al., 2019; Vinyals et al., 2019) over the past several years have led to artificial agents (AAs) capable of producing behavior that meets or exceeds human-level performance across a wide variety of tasks. Some notable advancements include DRL agents learning to play solo or multiagent video games [e.g., Atari 2,600 games (Bellemare et al., 2013), DOTA (Berner et al., 2019), Gran Turismo Sport (Fuchs et al., 2020), Starcraft II (Vinyals et al., 2019)], and even combining DRL with natural language processing (NLP) to win at text-based games such as Zork (Ammanabrolu et al., 2020). There have also been major developments in the application of DRL agents for physical systems, including applying DRL to automate a complex manufacturing-like process for the control of a foosball game table (De Blasi et al., 2021), using DRL to control a robotic agent during a collaborative human-machine maze-game (Shafti et al., 2020), and for the control of a robotic hand performing valve rotation and finger gaiting (Morgan et al., 2021). Recent research has also focused on implementing DRL agents in multiagent human-AA settings, including recent work in which a human teammate collaborated with unmanned aerial vehicles driven by DRL agents to douse fire in a real-time environment, where the task goals could not be achieved by either the human teammate or AAs alone (Navidi et al., 2020).

In DRL, agents learn via trial-and-error by modifying their behavior to maximize desired outcomes. Indeed, a primary advantage of using DRL techniques is that AAs can be trained and optimized to achieve task success across a variety of tasks without prior knowledge of the dynamics of the environment or the agent's action capabilities. However, research on DRL frequently lacks adequate discussion of the low-level dynamics of the behavior itself, and rather shifts its focuses on meta-level or global-level performance metrics (e.g., Mnih et al., 2015; Pohlen et al., 2018; Berner et al., 2019; Vinyals et al., 2019). In doing so, the current literature lacks perspective on the qualitative nature of AA behavior, leaving questions regarding the spatiotemporal patterning of their behavior largely unanswered. In fact, these agents often produce behavior that is easily distinguishable from or incompatible with human behavior, and for certain applications, this can be non-optimal. For instance, in the case of an AA acting alongside humans as an autonomous squad member (Chen et al., 2018), as a robot or virtual guide through a museum (Philippsen and Siegwart, 2003; Swartout et al., 2010), when training humans to perform a multiagent team task (Rigoli et al., 2020), or when navigating autonomously alongside humans in pedestrian areas (Carton et al., 2013) or on highways (Urmson et al., 2009; Wollherr et al., 2014; Weiss et al., 2015). Moreover, human-like movement or navigation is known to improve human-AA interaction in a myriad of ways, including increases in fault tolerance, trustworthiness, work pace, and perceived predictability (Carton et al., 2016, 2017; Castro-González et al., 2016; Obaid et al., 2016).

## Behavioral Dynamics of Human Route Selection

An alternative method for controlling AA behavior is to incorporate behavioral dynamics models (Warren, 2006; Warren and Fajen, 2008) and/or related task-dynamics models (Saltzman and Kelso, 1987) of human behavior within the control architecture of AAs. Consistent with the more general dynamical and complex systems approach to human behavior (Kugler et al., 1980; Haken et al., 1985; Thelen and Smith, 1994; Kelso, 1995, 2009; Richardson et al., 2014; Richardson and Kallen, 2015) the behavioral dynamics approach places a strong emphasis on *self-organization* and *contextual emergence*, whereby the organization or stable order of observed behavior is not understood to be the result of any individual system component, mechanism, or centralized control structure, but rather is understood to emerge as a posterior consequence of the distributed interaction of physical (lawful) processes, informational and biomechanical couplings, and contextual constraints. Accordingly, stable behavior is assumed to reflect a functional grouping of structural elements within an agent-environment system (e.g., limbs, movements and actions of actors, and objects and events of the task environment) that is temporarily constrained to act as a single coherent unit (also termed *synergy*), formed, and destroyed in response to changing task and sub-task goals and action possibilities (i.e., affordances).

Central to the approach is both identifying and then defining the self-organizing physical and informational constraints and couplings that underly the emergence of stable and effective human perceptual-motor behavior in the form of a non-linear dynamical system. Of particular relevance here is that, although identifying and defining such non-linear dynamical models may at first seem rather difficult, there is now a substantial body of research demonstrating how human perceptual-motor behavior can be modeled (and perhaps even derived) using a simple set of dynamical motor primitives (Haken et al., 1985; Kay et al., 1987; Schaal et al., 2005; Warren, 2006; Ijspeert et al., 2013; Richardson et al., 2015; Amazeen, 2018; Patil et al., 2020). Specifically, these dynamical motor primitives correspond to the fundamental properties of non-linear dynamical systems, namely (i) point-attractor dynamics and (ii) limit-cycle dynamics, with the former capable of capturing discrete movements or actions (e.g., tapping a key, passing, or throwing a ball) by means of environmentally coupled damped mass-spring functions, and the latter capable of capturing rhythmic movements (e.g., hammering, walking) by means of forced (driven) damped-mass spring systems or non-linear self-sustained oscillators (e.g., Rayleigh or van der Pol oscillator). In the context of modeling human perceptual-motor behavior, these dynamical primitives can be termed dynamical perceptual-motor primitives (DPMPs).

**Figure 1:**
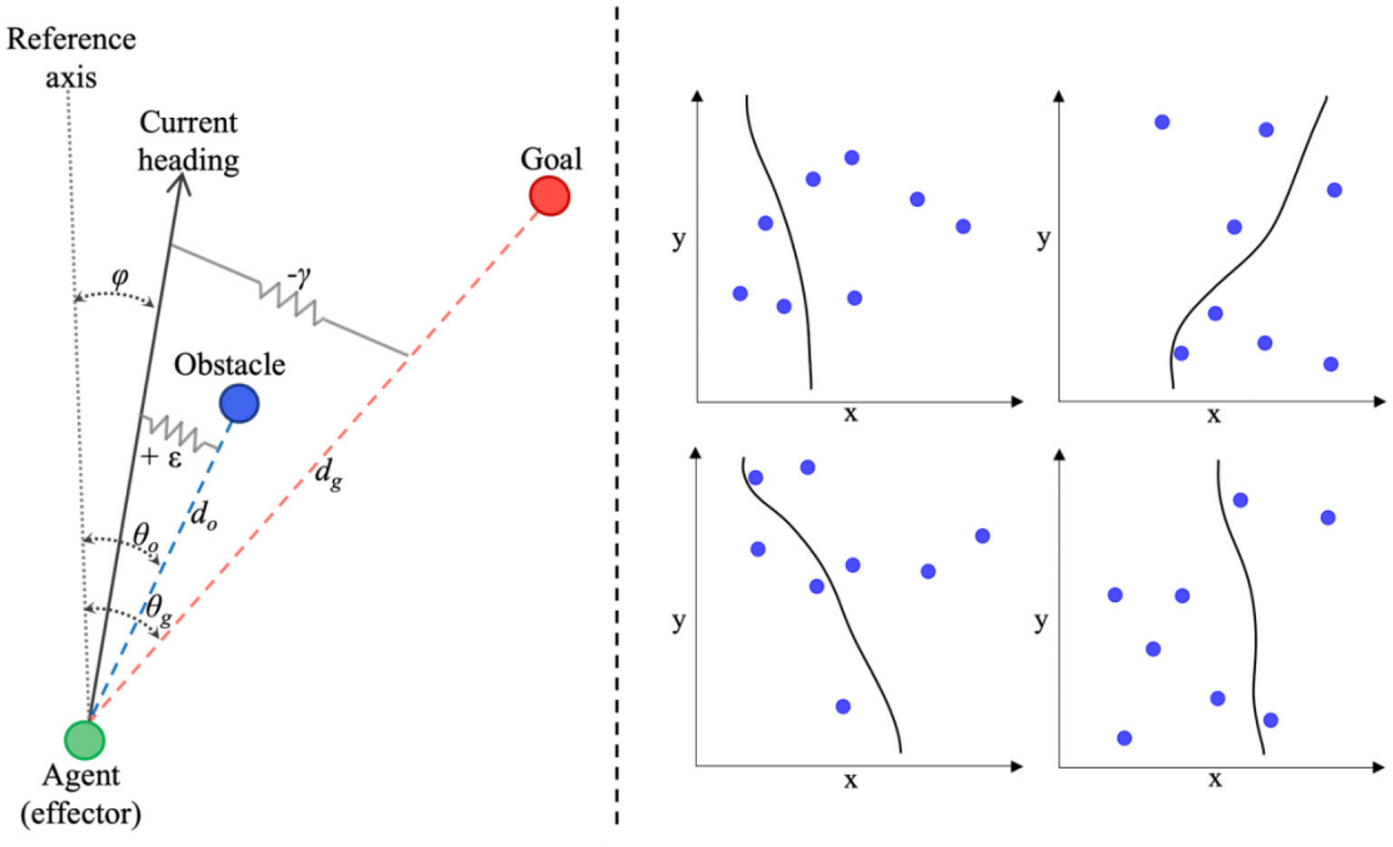
**(Left)** Abstract representation of the task-space devised by Fajen and Warren (2003) to capture the bearing behavior or heading direction, φ, of an agent attracted toward a target goal, g, while avoiding environmental obstacles, o. **(Right)** Four examples of route selection trajectories generated by the participant-specific parameterized version of the Fajen and Warren (2003) model (see text for more details about the model and model functions).

Dynamical perceptual-motor primitive models can successfully capture a wide range of human behaviors, from human object passing (Lamb et al., 2017), reaching, cranking, and rhythmic wiping (Kay et al., 1987; Saltzman and Kelso, 1987), intersegmental coordination (Amazeen et al., 1998), to drumming and racket ball tasks (e.g., Sternad et al., 2001; Ijspeert et al., 2013), as well as complex multiagent tasks (Dumas et al., 2014; Richardson et al., 2015; Nalepka et al., 2017, 2019; Lamb et al., 2019). One of the most prominent information-based models from this approach is the Fajen and Warren model for route selection and obstacle avoidance (hereafter referred to as the FW-DPMP model; Fajen et al., 2003 and Fajen and Warren, 2003, 2004), that employs simple, point attractive, and repulsive mass-spring-damper functions to model the changes in bearing or heading direction, φ, of an agent moving toward (attracted to) a goal location, while navigating around (repelled from) environmental obstacles. An abstract illustration of the task space used to define the FW-DPMP model is provided in Figure 1 (left) (adapted from Fajen and Warren, 2003; Warren and Fajen, 2008). In short, given a reference axis, *x*, in an (*x, y*) planar space, and assuming the agent is moving forward at a constant velocity, the FW-DPMP model defines the dynamics of an agent's heading direction, φ, to a target (goal) location, *g*, while avoiding obstacles, *o*_*i*_ using the equation


(1) $$\begin{array}{l} \ddot{\varphi }=-\beta \dot{\varphi }-\gamma \left(\varphi -\theta_{g}\right)\left(e^{-c_{1}d_{g}}+c_{2}\right)\\ +\sum_{i=1}^{\#obstacles}\varepsilon \left(\varphi -\theta_{O_{i}}\right)e^{-c_{3}\left|\varphi -\theta_{O_{i}}\right|}\left(e^{-c_{4}d_{O_{i}}}\right) \end{array}$$

Here, $\ddot{\varphi }$ represents the angular acceleration and $\dot{\varphi }$ represents the angular velocity of the agent's heading direction, θ_*g*_ represents the angle of the goal direction (with respect to the agent in an egocentric reference frame), θ_*O*_*i*__ represents the angle of an obstacle *i*, with *d*_*g*_ and *d*_*O*_*i*__ representing the distance to the target and obstacle *i*, respectively. The model parameters β, γ, and ε reflect the magnitude of damping or resistance toward changes in direction of heading, attraction to the direction of heading that leads to the goal location (i.e., positive stiffness toward the goal direction), and the repulsion force to repel the heading direction away from an obstacle location (i.e., negative stiffness toward obstacle direction), respectively. In other words, for a given value of β, steering toward a goal involves changing heading direction or turning rate, $\dot{\varphi }$, until φ − θ_*g*_ = 0 at a rate determined by γ, while avoiding or steering away from an obstacle involves a change in heading direction or turning rate, $\dot{\varphi }$, at a rate determined by ε away from φ − θ_*o*_ = 0. The fixed constants *c*_1_ and *c*_2_ influence the rate of decay of the attraction toward the goal and the scaling of the minimum acceleration (such that it never reaches zero, even for large goal distances), respectively, and *c*_3_ and *c*_4_ influence the angular acceleration away from obstacles and the rate of decay of the repulsion of obstacles, respectively.

The simple transparency with which Equation (1) can capture complex human steering behavior and locomotory navigation has been verified across numerous experimental procedures and tasks (see Warren, 2006; Warren and Fajen, 2008 for reviews). Modest extensions of Equation (1) can also capture patterns of collective behavior in crowd locomotor dynamics (Bonneaud et al., 2012; Warren, 2018), the dynamics of intercepting a moving target (Fajen and Warren, 2007), robot navigation (Huang et al., 2006) object pick-and-place tasks (Lamb et al., 2017), and more recently, navigation around obstacles while using a short-range haptic sensory substitution device (Lobo et al., 2019). Moreover, it is important to note that in each of the above studies, the movement trajectories generated by the model are not a result of a-priori computation (planning), but instead, are simply a situated self-organized result of interacting attractors and repellors.

## Current Study

The current study had two exploratory aims. The first was to investigate the degree to which the navigation and route selection trajectories of DRL agents (i.e., AAs trained using DRL) through an obstacle ridden environment were equivalent (and/or different) from those produced by human agents. The second and related aim was to determine whether the FW-DPMP model of human route navigation [i.e., Equation (1)] could not only be used to capture both human and DRL navigational behavior, but also help to identify whether differences between human and DRL trajectories were a function of different parametric tunings in the attractive and repulsive forces that shape an agent's movement toward a goal location while avoiding obstacles. Motivating the latter question was the possibility that one reason why DRL agents produce behavioral patterns different from humans is that DRL methods over-optimize (tune) AA behavior to a given task context (Hasson et al., 2020) and thus exhibit less variability and less flexible behavioral patterning than human agents. Given the powerful function approximation properties of deep neural networks (Csáji, 2001; Arulkumaran et al., 2017), examining the similitude with which the FW-DPMP model could capture both DRL and human navigation also provided a way of determining the degree to which the DRL and human agents learn to exhibit the same low-dimensional task dynamics.

To achieve these aims, human participants and DRL agents navigated a subset of 18 total obstacle configurations, each with two start locations and three goal/target locations, for a total of 108 unique combinations. Participants performed the task on a computer and used keyboard keys to control their movements through the environment. We examined (1) the similarities and differences between the navigational trajectories exhibited by human participants and the DRL agents and, after fitting the FW-DPMP model to the observed human and DRL agent trajectories, (2) whether different parametric tunings of the FW-DPMP model could account for any observed differences between the human and DRL agent trajectories.

Accordingly, the rest of the paper is structured as follows. First, we detail the experimental method used for data collection and the analysis methods employed to compare the observed human and DRL agent trajectories. We then report the results of this analysis. Following this, we detail the FW-DPMP model (Equation 1) fitting procedures and report a detailed analysis of the parametric differences between the simulated human and DRL agent trajectories. Note that additional analyses were employed to examine the degree to which the FW-DPMP model effectively captured the observed trajectories and to compare FW-DPMP-simulated human trajectories to simulated DRL agent trajectories, which can be found in the Supplementary Material.

## Experimental Methods

### Participants

Two hundred and five participants were recruited to participate remotely using Amazon Mechanical Turk (MTurk) for monetary compensation. MTurk participants ranged in age from 19 to 65 years old (M = 35.5, SD = 9.4), of which 32% were female and 68% were male. Twenty additional participants were recruited to participate in-person on Macquarie University campus for course credit or monetary compensation to validate the data collected remotely via MTurk (see section Confidence Interval Analysis for these comparative results, but in short, there were no differences between the groups). The in-person participants ranged in age from 18 to 35 (M = 23.6, SD = 5.3), of which 55% were female and 45% were male. One of the in-person participants and 13 Mturk participants were dropped from the analysis due to lack of task completion.

### Method and Apparatus

**Figure 2:**
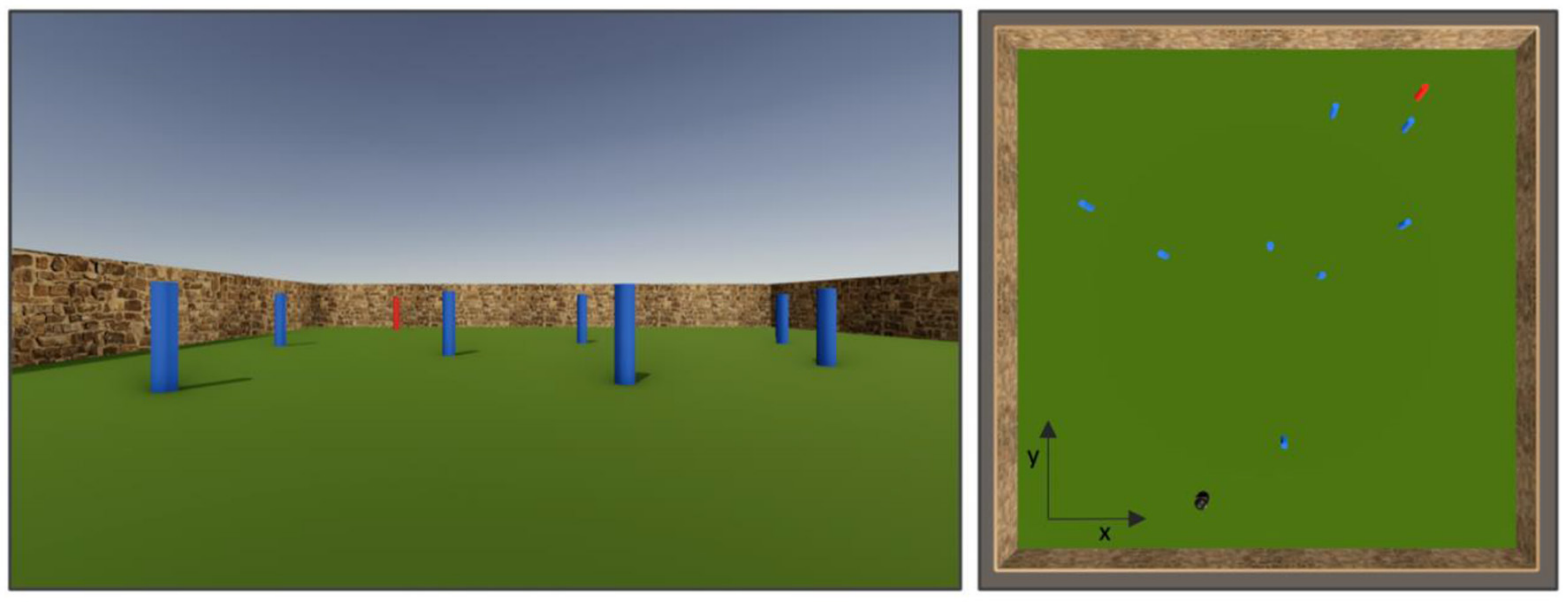
**(Left)** Depiction of the game field as seen by participants, with the target represented by the red cylinder and the obstacles by the blue cylinders. **(Right)** Top-down view of game field with reference axes, with the player represented by a black peg and the target represented by the red cylinder.

The route selection game and interface were developed using the Unity Game Engine (Unity Technologies, San Francisco, USA). As illustrated in Figure 2, the navigation environment was a 40 × 40 m square, surrounded by a “brick” textured wall, with the target (goal) location represented by a red cylinder and obstacles represented by blue cylinders (both measuring 0.5 m wide and 1 m tall). The game could be played on both PC and MAC computers using a web browser, with the playing area visualized by participants via a first-person camera view with a field of view of 60° in the vertical direction and 95° in the horizontal direction. Participants controlled their movements using a keyboard, with the left and right arrows used to control the heading direction (turn left and right) and pressing and holding down the spacebar to move forward along their current heading direction. When the space bar was pressed, the velocity of the participant's movements within the game environment was constant and was set to 10 m/s and the maximum rotation speed was set to 25° with an angular acceleration of 62.5°/s^2^. Participants were unable to move backwards but were able to stop moving (by releasing spacebar) and turn around completely (360°) using the arrow keys.

There were 18 randomly generated obstacle configurations. The positions of the obstacles for each configuration are included in the Supplementary Material. From a top down view of the playing area with (0, 0) at the center (see Figure 2), for each of these obstacle configurations, there were two starting positions (in meters): (5, −16) and (−5, −16,), which in practice corresponded to starting from either the left or right of the bottom of the field, and three target locations: (12, 16), (0, 16), or (−12, 16), which corresponded to the target being located at the top left, top middle, or top right of the field, respectively. In total, there were 108 combinations of the eighteen obstacle configurations, two starting locations, and three route types (18 × 2 × 3), where each unique combination is referred to as a “scenario.”

During a trial, if an obstacle was hit or bumped into, the trial would restart. A small counter was presented in the top right corner of the game displaying the number of successful trials completed. All participants began by completing five mandatory successful practice trials. Following this, the experiment required participants to complete 108 successful trials. Each participant was assigned a set of three obstacle configurations, which included the two starting positions and the three route types, resulting in 18 unique scenarios (3 × 2 × 2) per participant. Each scenario was repeated six times resulting in a total of 108 trials per participant which were presented in a random order.

Each of the in-person participants performed the task twice, once using a keyboard and once using a joystick (with the order counterbalanced). The joystick data was collected in order to compare the trajectories resulting from a continuous controller (joystick) to a discrete controller (keyboard). The joystick controls were as follows: shifting the joystick to the left or right moved the player in that direction and holding down the joystick's trigger moved the player forward. All MTurk participants completed the task using their own personal computer and keyboard. Both in-person and MTurk participants completed a Qualtrics survey before starting the task which collected basic demographics (i.e., age, gender, ethnicity, handedness). The survey also included a standardized set of instructions for how to play the game. Put shortly, participants were told to do their best to navigate to the red target object as quickly as possible, without hitting any obstacles. Lastly, participants were made aware that they would be paid the same amount regardless of the time taken to complete the experiment.

### DRL Agent Training

The same route selection game described above was used to train the DRL agents. Two types of DRL agents were trained to reach the target while avoiding obstacles: (1) using raycast data and (2) using visual pixel data as inputs. A raycast is conceptually like a laser beam that is fired from a point in space along a particular direction and any object that the beam hits can be detected, thus a set of N raycasts projected at the different angles from an agent's location can provide a discrete map of objects within the projected raycast region. Visual pixel data is simply screen capture data and is analogous to what the participants would see on the screen. The Unity MLAgents framework (Juliani et al., 2018) was used to train the agents whose policy was refined by the Proximal Policy optimization (PPO) methodology (Schulman et al., 2017) of RL. Proximal Policy optimization uses a combination of the Clipped Surrogate Objective function and multiple adaptive epochs on batches of state-action-reward combinations in order to update the policy of the agent, which in this case, is approximated by a deep neural network. Both types of agents were trained to generate five outputs corresponding to two one-hot “move” actions [move forward or do not move] and three one-hot “rotate” actions [rotate left, do not rotate, or rotate right].

The raycast agents were given inputs from 11 rays with one ray pointing straight ahead and five rays on each side spread equally over 45°. Each ray transmitted the information about what it was detecting in a one-hot vector of size 4 corresponding to whether it hits an object with one of the tags [one input each for target or obstacle], it hits an object with another tag [not a target or obstacle], and it does not hit anything, thus, resulting in an input size of 44. Furthermore, two additional inputs were given corresponding to the heading direction and distance of the target at any given time. The raycast agents' policy was controlled by a deep neural network consisting of an input layer with 46 neurons, two hidden layers with 128 neurons each, and an output layer with five outputs.

The visual agent was given an input from the first-person camera view (similar to the view presented to human participants) compressed to an RGB matrix of 80 × 144. The policy was controlled by a deep neural network consisting of two convolutional layers with kernel sizes 16 × 3 × 8 × 8 and 32 × 16 × 4 × 4, respectively, two hidden layers consisting of 512 neurons each, and an output layer with five outputs.

Both raycast and visual agents were trained for 5 million training steps. The hyperparameters used during training for the agent with raycast inputs were- batch size: 128, buffer size: 2,048, learning rate: 0.0005, beta: 0.01, epsilon: 0.2, lambda: 0.95, num epoch: 3, learning rate schedule: linear and for the agent with visual inputs were batch size: 128, buffer size: 10,240, learning rate: 0.0005, beta: 0.01, epsilon: 0.2, lambda: 0.95, num epoch: 3, learning rate schedule: linear (refer to Juliani et al., 2018 for more details). During each episode, which lasted either for 30 s or until the agent collided with an obstacle or the target, the obstacles were randomly placed in the playing area while the observations were collected every fifth frame and the environment updated at 50 Hz. The agent was given a (+1) reward for hitting the target and (−1) reward for either hitting an obstacle or not hitting anything within the trial duration. For the first 3 million training steps, the agent's start position and the target's position were randomly selected and for the final 2 million steps, the agent and the target were placed on the far sides of the playing area such that the x coordinates were −16 and 16, respectively, and the y coordinates were randomly selected. For the first 1.5 million training steps of the visual agent, the number of obstacles was reduced to four in order to facilitate learning by increasing the probability of a positive reward.

Twenty agents were trained by using the raycast and the target position as inputs and five agents were trained by using visual inputs. All the trained DRL agents were simulated to complete the task of reaching the target by presenting the same scenarios and using the same paradigms that were used for the human participants. Note that a third set of DRL agents was trained using raycast data as inputs, but without any target information, to determine whether they could learn the task completely from scratch and create the necessary dependencies on the task constraints. The results for these DRL agents and for the visual DRL agents are included in the Supplementary Material, but in short, both agents produced learning behavior that was nearly equivalent to the DRL agent with target information. Specifically, the DRL agents trained by visual data exhibited similar behavior to the agents with raycast and target position as inputs, whereas DRL agents trained by raycast data without the target information exhibited higher variability in their trajectories (refer to Supplementary Material), which can be partly attributed to the search behavior in the absence of direct visibility to the target and absence of information between raycasts. Given that human participants were always aware of the target location, they never exhibited such search behavior, and thus, comparing the variability of the DRL agents using raycast inputs without explicit target position information to the human trajectories could not be justified. Therefore, the raycast agents trained with target information (described above) were used as the DRL agents for the current study.

### Data Pre-processing

**Figure 3:**
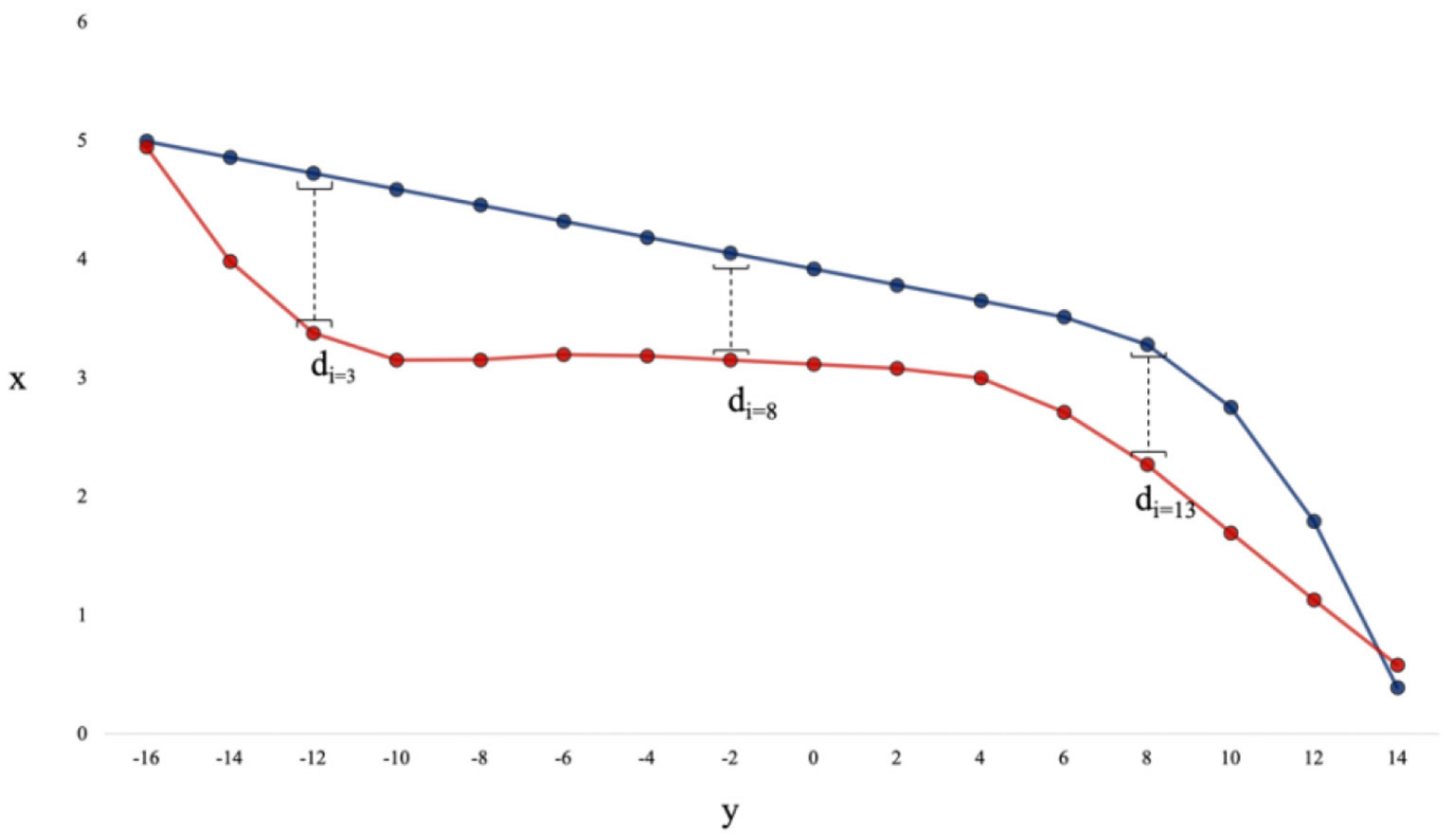
The distance between a pair of trajectories is the average of the absolute values of d_i_ calculated for every 20 cm bin along the y-axis. This example has 16 points per trajectory for illustration purposes, however the actual data had 160 points per trajectory.

The raw data for each trajectory (sampled at 50 Hz) were first interpolated to a standard-length of 1,000 data points. Any trajectory that was 1.2X longer in distance than the average trajectory length for that scenario (i.e., the average cumulative distance) was discarded, as these trajectories represented paths where a human or DRL agent took a highly anomalous path to the target. Further, any trajectory that took the participant more than 20 s to complete was also discarded, as the average trial time was only 6 s. Out of all trajectories, <5% were discarded. Following this, the value of each trajectory in the *x* coordinate was binned in intervals of 20 cm along the *y* axis (from −16 to 16 meters on the y axis), such that each trajectory had 160 points (see Figure 3 for illustration of this process). Analysis was done using Python 3.8, with larger computational tasks (e.g., agent training, model fitting) performed using the Multi-modal Australian Sciences Imaging and Visualization Environment (MASSIVE) high performance computing infrastructure (Goscinski et al., 2014) and the National Computational Infrastructure (NCI), which is supported by the Australian Government.

## Analysis

**Figure 4:**
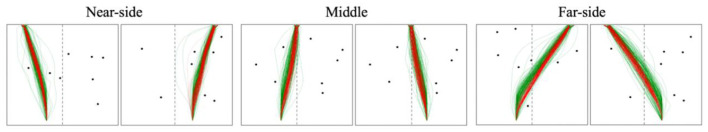
Example scenarios from the raw human (green) and DRL agent (red) data displaying the starting positions (i.e., left or right) and target position(s) (i.e., left, right or middle) for each route type.

To help simplify the analysis, the trajectories for each scenario were collapsed such that any trajectory with a starting location and target position on the same side (i.e., both on the left or both on the right) was classified as a *near-side* trajectory, any trajectory with a starting location and target position on opposite sides (i.e., starting on the left and target on the right, and vice versa) was classified as a *far-side* trajectory, and any trajectory with the target position in the middle was classified as a *middle* trajectory (note, preliminary analysis reaved the results were symmetrical with regards to route type, justifying this simplification). These three classifications are hereafter referred to as “route types” (see Figure 4). Further, in order to allow for comparison between trajectories, the data was pre-processed such that each value of a trajectory in the *x* coordinate was binned in intervals of 20 cm along the *y* axis, similar to Fajen and Warren (2003) and Lobo et al. (2019).

The proceeding analyses examine the differences between the human and DRL agent trajectories (both raw and simulated), as well as the differences between the near-side, far-side, and middle trajectories. In order to explore these data, three main analyses were employed: (i) number of preferred routes, (ii) confidence interval analysis, and (iii) the distance/error between trajectories. These analyses are described in detail in the following sections.

### Preferred Paths

**Figure 5:**
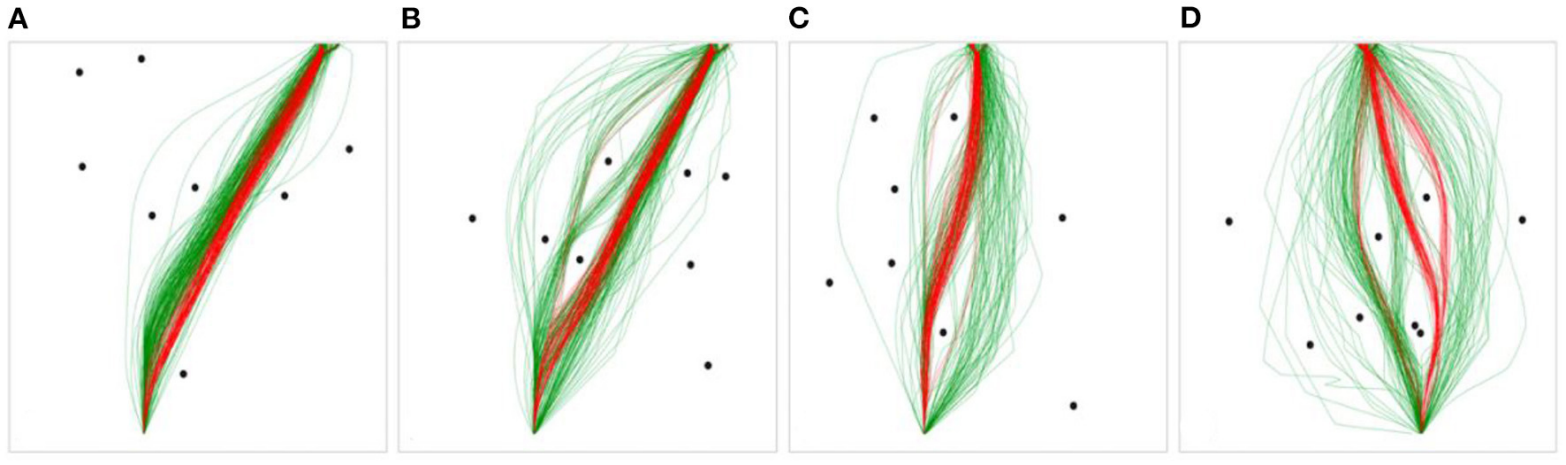
Example scenarios with human trajectories in green, and DRL agent trajectories in red. **(A)** contains one preferred path for humans and DRL agents, **(B)** contains three human preferred paths and one DRL agent preferred path, **(C)** contains two human preferred paths and one DRL agent preferred path, **(D)** contains three human preferred paths and two DRL agent preferred paths.

The number of preferred routes was examined as a global measure of the overall flexibility and variability in route selection displayed by the humans and DRL agents. To determine the number of routes taken by human participants and the DRL agents for each scenario, any route that contained at least 10% of the humans' or DRL agents' trajectories and that took a distinct path around an obstacle or obstacles was counted as a “preferred route” (see Figure 5 for examples of preferred routes). Preferred routes were determined by the researcher manually/by-hand.

### Confidence Interval Analysis

A confidence interval analysis was employed to determine the degree to which the mean DRL agent trajectory fell within the bounds of the 95% confidence interval (CI) of the human-produced trajectories, and thus provides a measure of the degree of similarity between the two groups. This was calculated by determining whether the mean trajectory of the DRL agents was in-between the bounds of the human 95% CI at each time-step and then calculating the proportion of time-steps where the mean DRL agent trajectory lay within the bounds. This was calculated separately for each of the 108 scenarios.

The mean DRL agent trajectories were calculated for each scenario by simply taking the mean *x*-value across all of the DRL agent-produced trajectories within each of the 20 cm bins along the *y*-axis. The upper and lower 95% CIs for the humans were calculated by taking the *x*-values at the 97.5th and 2.5th percentiles within each of the 20 cm bins along the *y*-axis, such that 5% of the human data points fell outside of the bounds for each bin. Following this, the code checked whether the mean DRL agent *x*-value for each bin was less than the upper-limit and greater than the lower-limit of the human CI. Finally, the proportion of time-steps where the DRL agent mean *x*-value was within these bounds was calculated.

The CI analysis was also utilized to help determine the similarity between the human trajectories collected via Amazon Mechanical Turk (MTurk) and the human trajectories collected in the lab. For each scenario, the proportion of the mean MTurk trajectory that fell within the 95% CI of the in-lab human trajectories was calculated. This was done separately for the joystick-control and keyboard-control data. The analysis revealed that 100% of the mean MTurk trajectory fell within both the keyboard-controlled and joystick-controlled human 95% CI for all scenarios, indicating that the mean MTurk trajectory was not significantly different from the in-lab participants (at the 5% level) across all scenarios. Thus, we proceeded with the MTurk data for all analyses in the paper. See Supplementary Figures 3, 4 in Supplementary Material for example scenarios displaying the mean trajectories and CIs.

### Distance Between Trajectories

The distance measure was calculated to provide a quantitative measure of the difference between trajectories to allow for comparisons between the human-produced and DRL agent-produced trajectories. Similar to Fajen and Warren (2003) and Lobo et al. (2019), distance was calculated by taking the average of the absolute value of the differences between the *x*-values of two trajectories within each 20 cm bin along the *y*-axis (see Figure 3 for an illustration of this process).

Distance measures were calculated between human and DRL agent trajectories within each scenario for each possible pairing of human- and DRL agent- produced trajectories. The measures were then averaged for each route type (i.e., near-side, middle, and far-side, see Figure 4) and across the 20 DRL agents for each human participant, such that the final analysis included three average distance measures for each route type and for each of the 192 human participants (e.g., participant 10's trajectories had an average distance of 2.69 cm across all DRL agents' trajectories for near-side routes, an average distance of 2.91 cm across all DRL agents' trajectories for middle routes, and an average distance of 3.70 cm across all DRL agents' trajectories for far-side routes).

Distance measures were also calculated within-groups (i.e., separately for humans and DRL-agents) to provide a measure of within-group variability, such that the distance was calculated for every possible pairing of two human-produced trajectories (excluding trajectories produced by the same participant) within each scenario, and then averaged for each route type and for each participant, such that the final analysis include three average distance measures for each route type and for each of the 192 participants (e.g., participant 10's trajectories had an average distance of 2.83 cm across all other human trajectories for near-side routes, an average distance of 3.74 cm across all other human trajectories for middle routes, and an average distance of 3.25 cm across all other human trajectories for far-side routes). The same method was used to calculate the distances for the DRL agents.

Finally, the same measures were calculated for the simulated data, and the corresponding results for these measures (including examining the degree to which the FW-DPMP model effectively captured the observed trajectories and comparing FW-DPMP-simulated human trajectories to simulated DRL agent trajectories) can be found in the Supplementary Material.

## Experimental Results

### Preferred Routes

For the human data, 41.67% of the scenarios contained greater than one preferred path, and 8.3% contained greater than two. No scenarios contained greater than three preferred paths. For the DRL agent data, 27.78% of the scenarios contained greater than one preferred path, and none contained greater than two. Further, 73.15% of the 108 total scenarios contained the same preferred path(s) for both humans and DRL agents, of which 26.58% contained two preferred paths. The scenarios in Figure 5 (excluding Figure 5A) were chosen to exemplify diversions from normality, where humans and DRL agents chose to take a variety of paths around the obstacles to reach the target location. However, the preferred routes chosen by humans and DRL agents tended to overlap across the majority of obstacle scenarios.

### Confidence Interval Analysis

**Figure 6:**
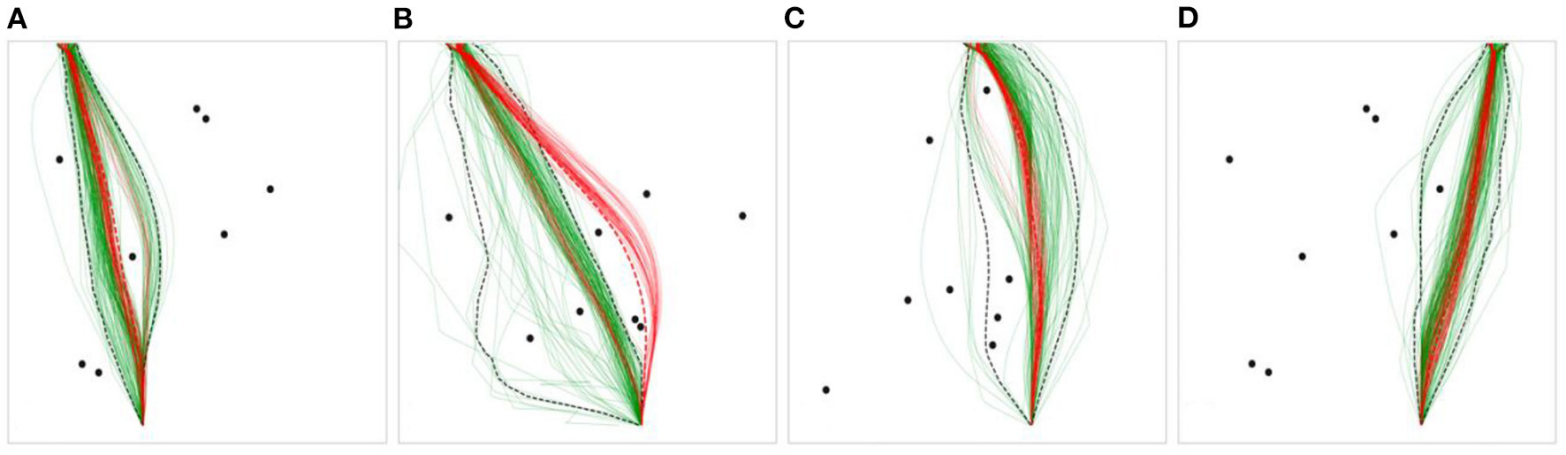
Example scenarios with human trajectories in green, DRL agent trajectories in red, human CIs in black-dash, and DRL agent mean trajectories in red-dash. Percent of the mean DRL agent trajectory contained in the human CI for each plot (from left to right): 91.9, 25.6, 100, 100%. **(A)** and **(B)** display rare examples of the mean DRL agent trajectory deviating from the human tendency, captured by the human CI, while **(C)** and **(D)** display more typical cases.

The CI analysis revealed that on average, 98.45% of the mean DRL agent trajectory fell within the human 95% CI, with a standard deviation of 9.08%. See Figure 6 for example scenarios displaying the mean trajectories and CIs. Only two out of 108 total scenarios contained DRL agent mean trajectories that were <91% inside the human CI. In one of these instances (displayed in Figure 6B) we see that the majority of DRL agents chose a distinct curved route around the obstacles compared to the humans, for whom the vast majority chose to navigate in a relatively straight line through the obstacles to the target location. Additionally, only five scenarios contained DRL agent mean trajectories that were between 91.25 and 97.5% inside the human CI, while the remaining 101 out of 108 total scenarios contained DRL agent mean trajectories that were >98% within the bounds of the human CI.

In summary, the analysis revealed a high degree of similarity between the DRL agent-produced and human-produced trajectories across all scenarios, such that the DRL agents tended to navigate through these environments in a manner that was relatively indistinguishable from human agents. However, as can be seen from inspection of Figure 6, at the group-level, DRL agents not surprisingly exhibited much less spread/variability across trajectories compared to humans.

### Distances Between Humans and DRL Agent Produced Trajectories

Distances (i.e., the average of the binned X-differences) were calculated between all pairs of DRL agent and human observed trajectories for each scenario in order to compare the trajectories produced by both groups (i.e., between human-produced and DRL agent-produced trajectories). This measure is hereafter referred to as Distance_(BG)_, where “BG” denotes a between-groups comparison.

A repeated measures ANOVA was conducted in order to examine whether there was a difference in Distance_(BG)_ for the three route types (near-side, middle, and far-side). The analysis revealed a significant main effect of route type, *F*_(2, 380)_ = 118.12, *p* < 0.001, η_*p*_^2^ = 0.38. *Post-hoc* analyses (pairwise comparisons) conducted using the Bonferroni correction revealed that Distance_(BG)_ for the far-side location (M = 1.8, SD = 0.8) was significantly higher than for the near-side location (M = 1.3, SD = 0.6), and middle location (M = 1.3, SD = 0.7; both *p* < 0.001), indicating that humans and DRL agents produced more dissimilar trajectories for the far-side routes, which with longer travel distance presented more opportunity for divergence in route selection around obstacles. However, the near-side location and middle location were not significantly different from each other, indicating that human and DRL agent trajectories remained relatively similar for these route types.

### Within-Group Distances

Distances were calculated between all possible pairings of trajectories within each scenario separately for DRL agents and Humans, and thus provides a measure of within-group variation.

To assess the effects of player type and route type on the binned X-differences, hereafter referred to as Distance_(WG)_, we defined a multilevel model predicting Distance_(WG)_, with fixed effects (*near-side, middle*, or *far-side*), and Player (*DRL agent* or *Human*), and random effect Player ID. In the model, independence of residuals was assumed but due to unequal variance between humans and DRL agents, we specified for the model to estimate distinct variance for each two player types (that is, the model estimated distinct variance for Distance_(WG)_ for *Human* trials and for *DRL agent* trials). The model was fit using the maximum likelihood estimation procedure.

This model adequately fit the data, $\chi_{\left(3\right)}^{2}$ = 484.62 *p* < 0.001. When the Player × Location two-way interaction was added to the model, the model again adequately fit the data, $\chi_{\left(5\right)}^{2}$ = 716.79, *p* < 0.001. The likelihood-ratio test of the two nested models was significant, $\chi_{\left(2\right)}^{2}$ = 217.11, *p* < 0.001, suggesting that the latter model fit the data more appropriately, and thus the latter model was adopted.

In the full model, there were significant main effects of Player, $\chi_{\left(1\right)}^{2}$ = 66.24, *p* < 0.001, and location, $\chi_{\left(2\right)}^{2}$ = 515.27, *p* < 0.001, and a significant Player × Location interaction, $\chi_{\left(2\right)}^{2}$ = 412.11, *p* < 0.001. Contrasts revealed that there were significant simple main effects of player on near-side location, $\chi_{\left(1\right)}^{2}$ = 61.55, *p* < 0.001, with humans (M = 1.6, SD = 0.6) having higher Distance_(WG)_ than DRL agents (M = 0.6, SD = 0.1); on middle location, $\chi_{\left(1\right)}^{2}$ = 29.26, *p* < 0.00, with humans (M = 1.3, SD = 0.7) having higher Distance_(WG)_ than DRL agents (M = 0.6, SD = 0.0); and on far side location, $\chi_{\left(1\right)}^{2}$ = 118.05, *p* < 0.001, with humans (M = 2.1, SD = 0.7) again having higher Distance_(WG)_ than DRL agents (M = 0.7, SD = 0.0). Thus, these results suggest that (not surprisingly) humans exhibited higher within-group variability in their trajectories as compared to DRL agents, who appeared to exhibit more consistency (i.e., less variation) across all the route types.

There was also a significant simple main effect of location for DRL agents, $\chi_{\left(2\right)}^{2}$ = 173.71, *p* < 0.001. Follow-up Bonferroni-corrected pairwise comparisons of Distance_(WG)_ for each location revealed that for the DRL agents, Distance_(WG)_ was significantly higher for the far-side location than both the middle location, *b* = 0.03, *t*_(19)_ = 3.57, *p* < 0.01, and the near-side location, *b* = 0.09, *t*_(19)_ = 12.77, *p* < 0.001. In addition, Distance_(WG)_ was higher for the middle location than for the near-side location, *b* = −0.07, *t*_(19)_ = 9.20, *p* < 0.001. Thus, DRL agents exhibited the highest degree of consistency in the near-side routes, and the highest degree of variability in the far-side routes.

Results also revealed significant simple main effect of location for humans, $\chi_{\left(2\right)}^{2}$ = 476.81, *p* < 0.001. Follow-up Bonferroni-corrected pairwise comparisons of Distance_(WG)_ for each of the locations revealed that for humans, Distance_(WG)_ was significantly higher for the far-side location than both the middle location, *b* = 0.74, *t*_(191)_ = 21.47, *p* < 0.001, and near-side location, *b* = 0.49, *t*_(191)_ = 14.15, *p* < 0.001. Contrary to the results for the DRL agents, Distance_(WG)_ for humans was significantly lower for the middle routes than for the near-side routes, *b* = −0.25, *t*_(191)_ = −7.29, *p* < 0.001. Thus, in contrast to DRL agents, humans exhibited the highest degree of consistency in the middle routes, and the highest degree of variability in the far-side routes.

Collectively, these results indicate that humans displayed higher variability overall [i.e., higher Distance_(WG)_] in their trajectories than DRL agents across all three route types, and both humans and DRL agents exhibited the most variation in the far-side routes. However, DRL agents were found to exhibit more variability in the middle routes than the near-side routes, while humans exhibited the opposite effect, such that there was higher human variation for the near-side routes than the middle routes.

## Model-Fitting and Simulations

### Model-Fitting

The FW-DPMP model was used to fit each of the observed trajectories produced by human participants and the DRL agents. For this study, the parameters γ, ε, and β were of central concern as these parameters are responsible for determining the goal's “attractiveness” and stability, repulsion from obstacles, and damping (which acts as a frictional force to oppose angular motion). These three parameters are ultimately responsible for the resulting/emergent trajectory. Thus, we fit the model to each trajectory by modulating these key parameters. For this study, all other parameters in Equation (1) were kept the same as inferred by the original Fajen and Warren (2003) formulation.

**Figure 7:**
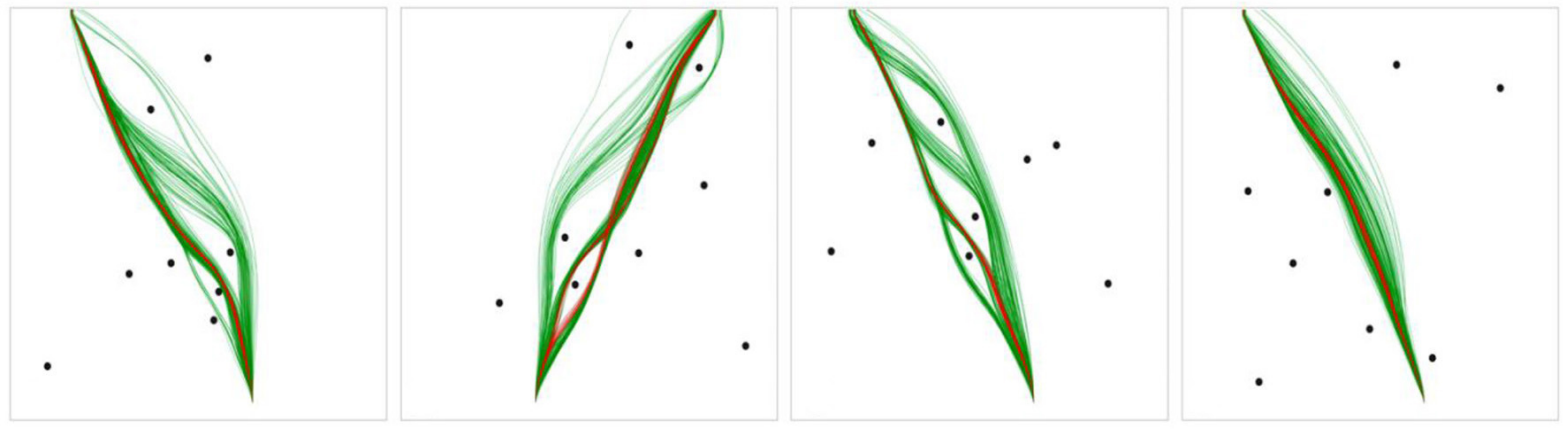
Example scenarios with human trajectories in green and DRL agent trajectories in red.

To determine the optimal γ, ε, and β parameters for a given human or DRL agent trajectory, we employed the differential evolution algorithm (Storn and Price, 1997) implemented in the SciPy.optimize package (Virtanen et al., 2020) which is a population-based search algorithm that can optimize a multivariate function by iteratively improving solutions based on an evolutionary process. In our case, the algorithm minimized the dynamic time warping (DTW) distance [implemented via the python similarity-measures package (Jekel et al., 2019)] between the observed trajectory and the simulated trajectory, using a range of parameter values until an optimal set was found that resulted in the smallest DTW error. Dynamic time warping is a method for measuring the similarity between two temporal sequences that may vary in speed/time by computing the distance between sequences using a one-to-many (and many-to-one) approach, such that the total distance between the sequences is minimized without concern for synchronization between the sequences. These parameters were then averaged for each human/DRL agent, such that each human/DRL agent ended up with their own characteristic set of parameters. The average, or characteristic parameters obtained for each agent were then used to simulate agent-specific movement trajectories within each scenario, resulting in a set of simulated trajectories for each human and DRL agent for all scenarios. See Figure 7 for examples of the simulated trajectories.

### Parameter Analysis

In order to determine what parameter estimates underlie the differences in the human and DRL agent parameter-defined model trajectories, we analyzed the differences in the parameters β, γ, and ε for humans and DRL agents across the three route types. Again, these parameters represent damping on turning rate, attraction to the goal, and repulsion from obstacles, respectively. Accordingly, we defined a multilevel model predicting parameter value with fixed effects Parameter (β, γ, or *transformed* ε), Route Type (*near-side, middle*, or *far-side*), and Player (*DRL agent* or *Human*), and random effect Player ID. The model was fit using the Maximum Likelihood estimation procedure. For readability, the simplified results are reported here; see Supplementary Material for the full details of this analysis. The means, standard deviations, minimum, and maximum values, and skew and kurtosis values for parameters β, γ, ε, and the Box-Cox-transformed ε are summarized in Table 1 of Supplementary Material.

The full three-way model (Parameter × Route Type × Player) adequately fit the data, $\chi_{\left(17\right)}^{2}$ = 35458.60, *p* < 0.001, and revealed significant main effects of parameter type, $\chi_{\left(2\right)}^{2}$ = 20591.37, *p* < 0.001, route type, $\chi_{\left(2\right)}^{2}$ = 80.32, *p* < 0.001, and player type, $\chi_{\left(1\right)}^{2}$ = 34.63, *p* < 0.001, on parameter value. There were also significant Parameter × Location, $\chi_{\left(4\right)}^{2}$ = 282.33, *p* < 0.001, Parameter × Player, $\chi_{\left(2\right)}^{2}$ = 125.59, *p* < 0.001, and Location × Player, $\chi_{\left(2\right)}^{2}$ = 74.22, *p* < 0.001, interaction effects on parameter value. Most importantly, the Parameter × Location × Player interaction was also significant, $\chi_{\left(4\right)}^{2}$ = 121.34, *p* < 0.001, suggesting that the Location × Player interaction varied for different parameter types. Contrasts were used to break down this interaction for each of the parameter types.

There were significant simple main effects of route type on β, $\chi_{\left(2\right)}^{2}$ = 159.50, *p* < 0.001, on γ, $\chi_{\left(2\right)}^{2}$ = 95.94, *p* < 0.001, and on *transformed* ε, $\chi_{\left(2\right)}^{2}$ = 260.96, *p* < 0.001. Similarly, there were significant simple main effects of player type on β, $\chi_{\left(1\right)}^{2}$ = 83.05, *p* < 0.001, on γ, $\chi_{\left(1\right)}^{2}$ = 47.77, *p* < 0.001, and on *transformed* ε, $\chi_{\left(1\right)}^{2}$ = 14.70, *p* < 0.001. Interestingly, there were also significant simple interaction effects of route type and player type on β, $\chi_{\left(2\right)}^{2}$ = 36.27, *p* < 0.001, on γ, $\chi_{\left(2\right)}^{2}$ = 80.92, *p* < 0.001, and on *transformed* ε, $\chi_{\left(2\right)}^{2}$ = 78.31, *p* < 0.001. These simple interaction effects suggest that the target-location simple main effects for each of the three parameter types was different for *human* and *DRL agent* players. These differing effects were further investigated using contrasts.

The results revealed significant simple main effects of route type on β for both *Human* players, $\chi_{\left(2\right)}^{2}$ = 21.74, *p* < 0.001, and *DRL agent* players, $\chi_{\left(2\right)}^{2}$ = 351.71, *p* < 0.001. Similarly, there were also significant simple main effects of route type on *transformed* ε for both *Human* players, $\chi_{\left(2\right)}^{2}$ = 15.36, *p* < 0.001, and *DRL agent* players, $\chi_{\left(2\right)}^{2}$ = 1354.99, *p* < 0.001. However, there was only a significant simple main effect of route type on γ for *DRL agent* players, χ^2^(2) = 448.61, *p* < 0.001. There was not a significant simple main effect on γ for *Human* players, $\chi_{\left(2\right)}^{2}$ = 3.80, *p* = 0.150. That is, γ-values did not significantly differ as a function of route type for human players.

Follow-up Bonferroni-corrected, pairwise comparisons of parameter value means for each of the parameter types for each of the route types and for each of the player types revealed that for DRL agents, γ-values were significantly lower when the target was on the same side as the starting position compared to when the target was in the middle, *b* = −49.57, 95% CI [−61.46, −37.68], *t*_(19)_ = −12.47, *p* < 0.001. Additionally, γ-values were also significantly lower when the target was on the far side to the player's starting position compared to when the target was in the middle, *b* = −81.65, 95% CI [−93.18, −70.12], *t*_(19)_ = 21.18, *p* < 0.001. γ-values for when the target was on the same side were significantly greater than values for when the target was on the far side, *b* = 32.08, 95% CI [21.51, 42.65], *t*_(19)_ = 9.08, *p* < 0.001.

For humans, β-values were significantly lower when the target was in the middle compared to when the target was on the far side to the player's starting position, *b* = −3.36, 95% CI [−5.56, 0.87], *t*_(181)_ = −4.57, *p* < 0.001. However, there were no significant differences in β-values when the target was on the same side as the player's starting position compared to when the target was in the middle, b = −2.42, 95% CI [−4.88, 0.04], *t*_(181)_ = −2.94, *p* = 0.059, or the far side, *b* = −0.95, 95% CI [−3.39, 1.50], *t*_(181)_ = −1.16, *p* > 0.999. For DRL agents, β-values were similarly significantly lower when the target was in the middle compared to when the target was on the far side to the player's starting position, *b* = −5.67, 95% CI [−6.98, −4.36], *t*_(19)_ = −12.92, *p* < 0.001. However, unlike the human players, DRL agents' β-values were significantly greater when the target was on the same side than when the target was on the far side, *b* = 2.34, 95% CI [1.16, 3.51], *t*_(19)_ = 5.96, *p* < 0.001, and when the target was in the middle, *b* = 8.00, 95% CI [6.72, 9.29], *t*_(19)_ = 18.62, *p* < 0.001.

For humans, *transformed* ε-values did not significantly differ between same-side and middle route types, *b* = −0.14, 95% CI [−0.33, 0.05], *t*_(181)_ = −2.23, *p* = 0.465, and did not significantly differ between same-side and far-side route types, *b* = 0.10, 95% CI [−0.09, 0.30], *t*_(181)_ = 1.6, *p* > 0.999. However, *transformed* ε-values were significantly lower for far-side route types than middle route types, *b* = −0.25, 95% CI [−0.43, −0.06], *t*_(181)_ = −3.90, *p* = 0.002. For DRL agents, *transformed* ε-values were also significantly lower for far-side route types than middle route types, *b* = −0.83, 95% CI [−0.89, −0.76], *t*_(19)_ = −36.31, *p* < 0.001, however *transformed* ε-values were also significantly lower in far-side-target trials than same-side-target trials, *b* = −0.27, 95% CI [−0.33, −0.21], *t*_(19)_ = −14.09, *p* < 0.001. Finally, *transformed* ε-values were significantly lower in same-side trials than in middle trials, *b* = −0.56, 95% CI [−0.63, −0.48], *t*_(19)_ = −21.17, *p* < 0.001.

**Figure 8:**
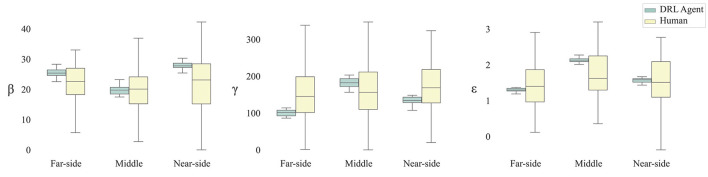
Box plots of parameter estimates for β, γ, and Box-cox corrected ε (i.e., damping, attraction to target, and repulsion from obstacles, respectively) by route type and by player.

Overall, and not surprising given the analysis of the raw data trajectories above, there was much less variability in the parameter estimates for DRL agents than for humans across all route types, reflecting the fact that the DRL agents were more finely tuned to changes in route type. Humans, on the other hand, tended to exhibit a large degree of variation in their trajectories, which is reflected in the wide range of parameter estimates for their data. This can be seen from an inspection of Figure 8. The results revealed that damping of turning rate (i.e., β) was lowest for the middle routes for both humans and DRL agents. However, damping was also lower for far-side routes than for near-side routes for DRL agents, but not for humans. Furthermore, there was no effect of route type on attraction to the target (i.e., γ) for humans, while for DRL agents, attraction was significantly different across all route types, such that attraction was highest for middle routes and lowest for far-side routes. Finally, repulsion from obstacles (i.e., ε) was lower for far-side routes than middle routes for both humans and DRL agents, but unlike humans, repulsion for DRL agents was also lower for far-side routes than near-side routes, and lower for near-side routes than middle routes.

## Discussion

The analysis of the trajectories produced by DRL and human agents revealed a high degree of similarity across all scenarios. That is, the DRL agents tended to navigate through the environments in a manner that was relatively indistinguishable from human agents. Specifically, the preferred routes chosen by DRL agents and humans tended to overlap across most scenarios, and, on average, 98.5% of the mean DRL trajectory fell within the human 95% CI. However, the analysis of observed trajectories also revealed that humans exhibited much more variability in their trajectories across all three route types than the DRL agents, which is clear upon visual observation of the trajectories produced by both groups (see Figures 5, 6 for examples). Consistent with this latter finding, the comparative analysis of the parameter estimates of β, γ, and ε, which represent damping on turning rate, attraction to the goal, and repulsion from obstacles, respectively, revealed more highly tuned parameter estimates for the DRL-agents compared to the human agents.

The parameter analysis also revealed similarities between the two groups in that repulsion was highest and damping was lowest for middle routes for both DRL and human agents, reflecting how for the shortest-distance routes, with less time and distance to allow for smooth adjustments in their heading, both groups steered away from obstacles abruptly. Further, DRL and human agents both exhibited low repulsion and higher damping for the far-side routes, indicating that for far-side routes (which on average involve the greatest travel distance) agents had more time to steadily adjust the angle of approach in their trajectory, such that they could smoothly avoid obstacles in the way of reaching the target, and could even closely shave (without hitting) obstacles on the way given the opportunity for finer control. Regarding the virtual nature of the task, a study by Fink et al. (2007) compared human paths while walking to a stationary goal and avoiding a stationary obstacle in matched physical and virtual environments and found small, but reliable, differences in locomotor paths, with a larger maximum deviation, larger obstacle clearance, and slower walking speed in the virtual environment. They concluded these differences were most likely the result of greater uncertainty regarding the egocentric location of virtual obstacles. However, they found that the trajectories from both environments had similar shapes with no difference in median curvature and could be modeled with a single set of DPMP parameter values, thus justifying the use of virtual environments to study locomotor behavior.

On one hand, given that a simple DPMP model (FW-DPMP model; Fajen and Warren, 2003, 2004) can capture the navigational behavior of human agents in an environment with obstacles, and that neural networks are powerful functional approximators (Csáji, 2001), it is perhaps unsurprising that the DRL agents produced trajectories that are consistent with those produced by the human participants. Indeed, the current findings suggest that the policies (state-action mappings) that the DRL agents learned during training approximated the same low-dimensional dynamics expressed by the FW-DPMP model (Equation 1); and, in turn, the DRL agents produced trajectories that closely matched the prototypical or near-optimal human (hyper-tuned) trajectories and human-parameterized FW-DPMP model trajectories. Indeed, one advantage of DRL methods for AA development is the capacity of deep neural networks to learn low-dimensional feature representations from high-dimensional state-action spaces (Arulkumaran et al., 2017). Thus, within the context of relatively simple perception-action tasks with a singular and easily defined task goal (and reward structure), like the virtual navigation task explored here, the similarity of DRL and human behavior is much more likely.

On the other hand, for more complex tasks that involve multiple sub-goals or equifinal possibilities to achieve task success, the highly tuned, near-optimal dynamics of DRL policies is also their downfall in that these policies can quickly and significantly diverge from those preferred (Carroll et al., 2019) or even attainable by human actors (Fuchs et al., 2020). This results in DRL agent behavior that is either incompatible or non-reciprocal with respect to human behavior (Carroll et al., 2019), or difficult for humans to predict (Shek, 2019), even requiring the human user to be more-or-less enslaved to the behavioral dynamics of the DRL agent to achieve task success (Shah and Carroll, 2019). Moreover, consistent with the current findings, even when the DRL agent policies and behavior are within the action landscape of human behavior (as was the case here), the over-tuned nature of DRL behavioral policies are often unable to capture the robust variability and flexibility of human behavior, requiring the implementation of rather complex and bespoke reward structures to cultivate different “types” of agents or DRL “personalities” (e.g., over avoidant or under-avoidant navigational agents).

The obvious advantage of DPMP models, with regard to the latter point, is that such models can not only be used to approximate human behavior across a wide range of simple and complex task contexts (Sternad et al., 2001; Ijspeert et al., 2013; Dumas et al., 2014; Lamb et al., 2017; Nalepka et al., 2017), but by making simple and transparent changes to model parameters one can also capture the variability of human behavior. Accordingly, the action dynamics of AAs controlled by DPMP models can be easily tuned or varied to capture various “types” of human actors or actor task preferences within (or even across) task contexts. However, defining the environmental couplings (information control laws) and functional details of DPMP models of human behavior does have its own challenges and often requires having a comprehensive a-priori understanding of the task-dynamics in question, which itself can require a significant degree of experimentation and trial and error. This is of course in stark contrast to the majority of contemporary DRL methods that do not require any knowledge of a task's underlying dynamics.

Given the complementary challenges associated with employing DPMP and DRL methods for the development of human-like AAs, Patil et al. (2021) have recently suggested employing a hybrid DPMP-DRL approach, allowing the DRL model to learn the high-level decision making while leaving the action dynamics to be captured by the DPMP functions. In summary, Patil et al. (2021) discovered that hybrid DPMP-DRL agents outperformed a heuristically parameterized DPMP model in terms of effectively representing characteristic expert human behavior. Indeed, combining DPMP and DRL model architectures can provide the best of both worlds by harnessing the generative “human-like” action patterns provided by DPMPs with the flexibility afforded by deep neural networks. In addition to augmenting DRL architectures by DPMP models, Patil et al. (2021) also argue that DPMP models can also be used to create synthetic datasets for training DRL agents with imitation learning (Bain and Sammut, 1999), which can provide scaffolding during the initial training steps of the DRL agents.

Finally, it is important to appreciate that one of the issues motivating the present research was the fact that the vast majority of DRL studies only focus on overall/global performance outcomes of DRL training, with little attention to the spatiotemporal patterning of the behavior enacted. Although the current study revealed that for a simple navigation task DRL agents produced behavior that appeared human-like in terms of its spatiotemporal patterning, this equivalence still highlights the importance of making such low-level behavioral comparisons. Indeed, as more and more Artificial Intelligence technologies start relying on DRL as a way of creating AAs that act alongside or replace human co-actors in complex tasks, it is crucial that the compatibility of the low-level, spatiotemporal behavior of AAs for human-AA interaction is assessed and not eclipsed by a desire for “super-human” performance levels. This is particularly important for social or multiagent tasks, where achieving greater than expert level performance does not necessarily lead to AAs capable of robust or even effective human interaction and coordination (Carroll et al., 2019), nor does it translate to AAs (acting as trainers) being able to induce optimal behaviors in novice humans (Rigoli et al., 2021).

## Data Availability Statement

The datasets presented in this study can be found in online repositories. The names of the repository/repositories and accession number(s) can be found at: https://github.com/Complexity-in-Action/Route-Navigation; https://osf.io/a67uf/.

## Ethics Statement

The studies involving human participants were reviewed and approved by Institutional Review Board at Macquarie University. The patients/participants provided their written informed consent to participate in this study.

## Author Contributions

LR, GP, MR, and RK: conceptualization. LR, GP, and MR: experiment design and setup, DRL agent training, and data collection. LR, HS, GP, and MR: data analysis and writing. MR and RK: funding acquisition and supervision. All authors contributed to the article and approved the submitted version.

## Funding

This work was supported by ARC Future Fellowship (MR, FT180 100447) and the International Macquarie University Project Specific Scholarship (LR, no. 2017659).

## Conflict of Interest

The authors declare that the research was conducted in the absence of any commercial or financial relationships that could be construed as a potential conflict of interest.

## Publisher's Note

All claims expressed in this article are solely those of the authors and do not necessarily represent those of their affiliated organizations, or those of the publisher, the editors and the reviewers. Any product that may be evaluated in this article, or claim that may be made by its manufacturer, is not guaranteed or endorsed by the publisher.
