# Supplementary material for: Navigational Behavior of Humans and Deep Reinforcement Learning Agents
Source: Front Psychol. 2021 Sep 22;12:725932. doi: 10.3389/fpsyg.2021.725932 (PMC8493935; doi:10.3389/fpsyg.2021.725932)
Supplement: Supplementary file 1 [file Table_1.DOCX]

Supplementary Material

# PARAMETER ANALYSIS

Shapiro-Wilk tests for normally distributed data were significant for parameters *b* (*W* = 0.98, *p* < .001), *γ* (*W* = 0.98, *p* < .001), and *ε* (*W* = 0.83, *p* < .001) suggesting that there were non-trivial deviations from the normal distribution for each of the parameters. However, the Shapiro-Wilk test is very powerful, especially for large sample sizes such as these (Rochon et al., 2012). Histograms and Q-Q plots for each variable suggested that only *ε* showed a meaningful deviation from a normal distribution. Further, *ε* appeared to be lognormally distributed.

To estimate the most suitable *λ* value for a Box-Cox (Box & Cox, 1964) transformation of *ε*, we fit a standard, left-hand-side model of *ε* predicted by player type and target location using Maximum Likelihood estimation, *χ*^2^(2) = 5.82, *p* = .054. In this model, *λ* was estimated to equal -.041 (*z* = -.84, *p* = .402). When *ε* was taken to the power of *λ*, the skewness of its distribution was reduced from 1.78 to .006 and kurtosis was reduced from 7.83 to 2.41. A Shapiro-Wilk test of the transformed distribution was still significant, but indicated a .16 increase in *W, W* = 0.99, *p* < .001.

Unsurprisingly, given each parameter type had completely different ranges, all Mauchly tests of homoscedasticity of parameter values across parameter types for each target location, each player type, and each combination of target location and player type were significant at the .001 significance level. Further, all Mauchly tests of homoscedasticity of parameter values across target locations for each parameter type, each player type, and each combination of parameter and player type were also significant, albeit at the .05 significance level, except for DRL-agent’s *b*, *γ*, and *ε* values. All Levene tests of homoscedasticity of parameter values across player types for each parameter type, each target location, and each combination of parameter type and target location were significant at the .001 significance level except for the test for values when the target was on the same side as the starting location, which was significant at the .01 significance level, and the test for values when the target was in the middle, which was not significant (*p* = .240). These results indicate that the assumptions of sphericity and homogeneity of variance between groups were violated.

These violations meant that a MANOVA would not be appropriate. Instead, to assess the effects of player type and target location on the calculated values of each of the three parameters, we defined a multilevel model predicting parameter value with fixed effects Parameter (*b*, *γ*, or *transformed ε*), Location (*near-side*, *middle*, or *far-side*), and Player (DRL-agent or *Human*), and random effect Player ID. In the model, independence of residuals was assumed but since there was unequal variance between parameter types, target locations, and between humans and DRL-agents, we specified for the model to estimate distinct variance for each of the 18 levels of the parameter-location-player fixed-effects combination (that is, the model estimated distinct variance for parameter *β* values calculated in *Human* trials when the target was on the same side as the starting location, for parameter *γ* values calculated in *DRL-agent* trials when the target was in the middle, etc.). The model was fit using the Maximum Likelihood estimation procedure.

This model adequately fit the data, *χ*^2^(5) = 10266.44, *p* < .001. When the Parameter × Location, Parameter × Player, and Location × Player two-way interactions were added to the model, the model again adequately fit the data, *χ*^2^(13) = 29295.03, *p* < .001. However, a likelihood-ratio test of the two nested models was significant, *χ*^2^(9) = 243.93, *p* < .001, suggesting that the latter model fit the data more appropriately.

Similarly, when the Parameter × Location × Player three-way interaction was added to the two-way model, the resulting three-way model also adequately fitted the data, *χ*^2^(17) = 35458.60, *p* < .001. The likelihood-ratio test of the two- and three-way models was significant, *χ*^2^(4) = 116.56, *p* < .001, and so the full, three-way model was adopted.

## Descriptive Statistics of Parameters

| Table 1. *Descriptive statistics for parameters β, γ,* and *ε.* | | | | | | | | | |
| --- | --- | --- | --- | --- | --- | --- | --- | --- | --- |
| Parameter | Player | Route type | n | Mean | SD | Minimum | Maximum | Skewness | Kurtosis |
| *β* | Human | Near-side | 183 | 21.53 | 8.59 | 0.01 | 49.41 | -0.29 | 2.93 |
|  |  | Middle | 183 | 19.11 | 7.11 | 0 | 37.61 | -0.50 | 3.30 |
|  |  | Far Side | 183 | 22.47 | 6.97 | 0.23 | 48.63 | 0.01 | 4.31 |
|  |  | Combined | 549 | 21.04 | 7.71 | 0 | 49.41 | -0.24 | 3.51 |
|  | DRL-agent | Near-side | 20 | 27.83 | 1.24 | 25.40 | 30.29 | -0.12 | 2.81 |
|  |  | Middle | 20 | 19.82 | 1.53 | 17.46 | 23.22 | 0.49 | 2.58 |
|  |  | Far Side | 20 | 25.49 | 1.30 | 22.53 | 28.30 | 0.06 | 3.15 |
|  |  | Combined | 60 | 24.38 | 3.64 | 17.46 | 30.29 | -0.43 | 1.88 |
| *γ* | Human | Near-side | 183 | 170.84 | 64.99 | 19.80 | 397.46 | 0.32 | 3.29 |
|  |  | Middle | 183 | 161.90 | 77.60 | 0.00 | 400.00 | 0.49 | 3.41 |
|  |  | Far Side | 183 | 156.91 | 75.11 | 1.05 | 355.37 | 0.55 | 2.89 |
|  |  | Combined | 549 | 163.24 | 72.85 | 0.00 | 400.00 | 0.44 | 3.20 |
|  | DRL-agent | Near-side | 20 | 133.40 | 11.89 | 107.06 | 148.16 | -0.67 | 2.56 |
|  |  | Middle | 20 | 182.97 | 13.83 | 156.18 | 203.15 | -0.02 | 1.93 |
|  |  | Far Side | 20 | 101.32 | 11.03 | 86.07 | 133.00 | 1.02 | 4.40 |
|  |  | Combined | 60 | 139.23 | 35.97 | 86.07 | 203.15 | 0.28 | 1.82 |
| *ε* | Human | Near-side | 183 | 9.25 | 7.42 | 0.64 | 33.42 | 1.26 | 3.89 |
|  |  | Middle | 183 | 10.78 | 8.58 | 0.80 | 60.00 | 1.93 | 9.13 |
|  |  | Far Side | 183 | 8.14 | 7.16 | 1.15 | 40.26 | 1.85 | 6.77 |
|  |  | Combined | 549 | 9.39 | 7.81 | 0.64 | 60.00 | 1.75 | 7.45 |
|  | DRL-agent | Near-side | 20 | 6.98 | 0.73 | 5.83 | 8.98 | 0.76 | 3.97 |
|  |  | Middle | 20 | 14.35 | 1.75 | 10.46 | 18.51 | 0.23 | 3.83 |
|  |  | Far Side | 20 | 4.93 | 0.32 | 4.28 | 5.38 | -0.39 | 2.30 |
|  |  | Combined | 60 | 8.76 | 4.22 | 4.28 | 18.51 | 0.73 | 1.98 |
| *Box-cox-corrected* *ε* | Human | Near-side | 183 | 1.54 | 0.63 | -0.37 | 2.77 | -0.05 | 2.34 |
|  |  | Middle | 183 | 1.68 | 0.60 | -0.19 | 3.20 | -0.10 | 2.63 |
|  |  | Far Side | 183 | 1.44 | 0.61 | 0.11 | 2.90 | 0.29 | 2.17 |
|  |  | Combined | 549 | 1.56 | 0.62 | -0.37 | 3.20 | 0.03 | 2.31 |
|  | DRL-agent | Near-side | 20 | 1.57 | 0.08 | 1.43 | 1.77 | 0.42 | 3.30 |
|  |  | Middle | 20 | 2.12 | 0.09 | 1.89 | 2.32 | -0.30 | 3.98 |
|  |  | Far Side | 20 | 1.30 | 0.05 | 1.19 | 1.37 | -0.50 | 2.45 |
|  |  | Combined | 60 | 1.66 | 0.36 | 1.19 | 2.32 | 0.42 | 1.65 |

# SIMULATED DATA

## Preferred Paths

For the simulated human data, 25.93% of the scenarios contained greater than one preferred path, and 4.63% contained greater than two. For the simulated DRL agent data, 5.56% (i.e., 6 out of 108 scenarios) of the scenarios two preferred paths, and none contained greater than two. Further, 76.85% of the 108 total scenarios contained the same number of preferred paths for humans and DRL agents, and of the six DRL agent scenarios that contained two preferred paths, four of them overlapped with the human scenarios containing two preferred paths. The scenarios in Fig. S1 (excluding Fig. S1d) were chosen to exemplify diversions from similitude, where humans and DRL agents chose to take a variety of paths around the obstacles to reach the route type. However, similar to the raw data presented in the main paper, the preferred routes for the simulated human and DRL agent data tended to overlap across the majority of obstacle scenarios.


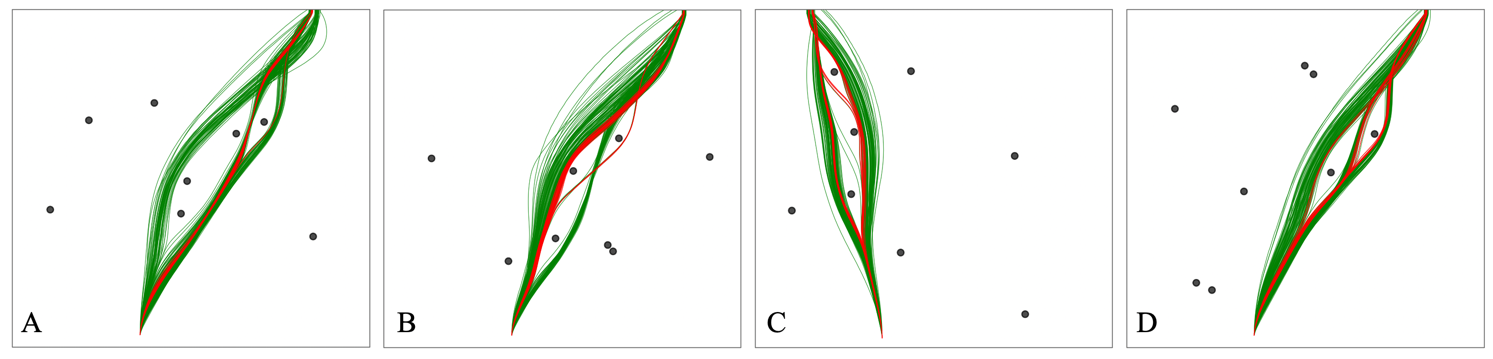


**Figure S1.** Example scenarios with human trajectories in green, and DRL agent trajectories in red. A) contains three human preferred paths and one DRL agent preferred path, B) contains two human preferred paths and one DRL agent preferred path, C) contains two human preferred paths and two DRL agent preferred paths, D) contains three human preferred paths and two DRL agent preferred path.

## Confidence Interval Analysis

The analysis revealed that ﻿on average, ﻿99.57% of the mean simulated DRL agent trajectory fell within the simulated human 95% CI, with a standard deviation of ﻿0.71, and a range of 97.5% - 100%. See Fig. S2 for example scenarios displaying the mean trajectories and confidence intervals. Overall, like the results for the raw data presented in the main paper, the analysis revealed a high degree of similarity between the simulated DRL agent and simulated human trajectories across the set of scenarios, such that the simulated DRL agent trajectories tended to navigate through these environments in a manner that was relatively indistinguishable from the simulated human trajectories (aside from differences in overall variability).


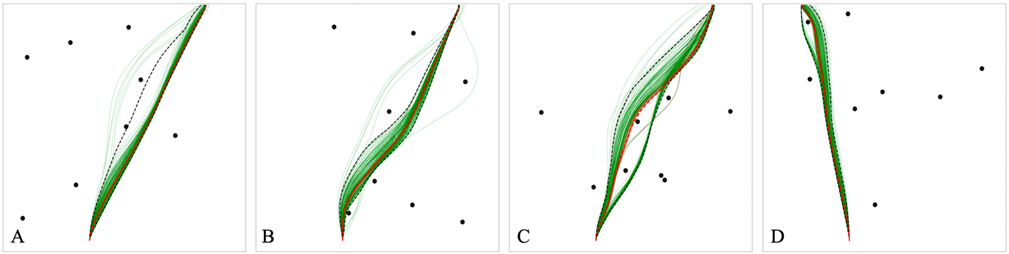


**Figure S2.** Example scenarios with human trajectories in green, DRL agent trajectories in red, human CIs in black-dash, and DRL agent mean trajectories in red-dash. The percent of the mean DRL agent trajectory contained in the human CI for each plot (from left to right): 98.7%, 100%, 98.8%, 100%

## Distances Between Humans and DRL Agents

Similar to the observed trajectories, the binned X-differences were calculated between the DRL agent and human simulated trajectories for each scenario in order to compare the trajectories produced by both groups, hereafter referred to as Distance_(S-BG)._ A repeated measures ANOVA was conducted to examine whether there was a difference in Distance_(S-BG)_ for the three route types (near-side, middle, and far-side). The analysis revealed a significant main effect of route type, *F*(2,380) = 118.12, *p* < .001, *η_p_² =* .38. Post hoc analyses (pairwise comparisons) conducted using the Bonferroni correction revealed that Distance_(S-BG)_ for the far-side location (M = 1.83, SD = 0.75) was significantly higher than for the near-side location (M = 1.29, SD = 0.64) and middle location (M = 1.27, SD = 0.70; both *p* < .001). However, the near-side location and middle location were not significantly different from each other. Thus, like the results for the raw data presented in the main paper, the difference/distance between the simulated human and simulated DRL agent trajectories was largest for the far-side location routes, and smaller for the near-side and middle routes.

## Within-Group Distances

Like the observed trajectories, the distances (binned X-differences) were calculated between all possible pairings of simulated trajectories within each scenario separately for DRL agents and Humans, and thus provided a measure of within-group variation, hereafter referred to as Distance_(S-WG)_. To assess the effects of player type and route type on Distance_(S-WG)_, we defined a multilevel model predicting parameter value with fixed effects (*near-side*, *middle*, or *far-side*), and Player (*DRL agent* or *Human*), and random effect Player ID. In the model, independence of residuals was assumed but since there was unequal variance between humans and DRL agents, we specified for the model to estimate distinct variance for each two player types (that is, the model estimated distinct variance for Distance_(S-WG)_ for *Human* trials and for *DRL agent* trials). The model was fit using the Maximum Likelihood estimation procedure.

This model adequately fit the data, *χ*^2^(3) = 2005.69, *p* < .001. When the Player × Location two-way interaction was added to the model, the model again adequately fit the data, *χ*^2^(5) = 2337.63, *p* < .001. The likelihood-ratio test of the two nested models was significant, *χ*^2^(2) = 235.92, *p* < .001, suggesting that the latter model fit the data more appropriately, and thus the latter model was adopted.

In the full model, there were significant main effects of Player, *χ*^2^(1) = 99.21, *p* < .001, and location, *χ*^2^(2) = 2211.35, *p* < .001, and a significant Player × Location interaction, *χ*^2^(2) = 1374.98, *p* < .001. Contrasts revealed that there was a significant simple main effect of player on near-side location, *χ*^2^(1) = 52.22, *p* < .001, with humans (M = 0.39, SD = 0.14) having higher Distance_(S-WG)_ than DRL agents (M = 0.07, SD = 0.01). There was also a significant simple main effect of player on middle location, *χ*^2^(1) = 40.11, *p* < .00, with humans (M = 0.29, SD = 0.13) having higher Distance_(S-WG)_ than DRL agents (M = 0.04, SD = 0.01), and finally a significant simple main effect of player on far side location, *χ*^2^(1) = 253.21, *p* < .001, with humans (M = 0.72, SD = 0.29) again having higher Distance_(S-WG)_ than DRL agents (M = 0.10, SD = 0.03). Thus, this analysis revealed results consistent with that of the raw/non-simulated data, such that the simulated human data exhibited higher within-group variability as compared to the simulated DRL agent data, which again exhibited more consistency/less variation across all the route types.

There was also a significant simple main effect of location for the simulated DRL agent data, *χ*^2^(2) = 252.42, *p* < .001. Follow-up Bonferroni-corrected pairwise comparisons of Distance_(S-WG)_ for each of the locations revealed that for the DRL agents, Distance_(S-WG)_ was significantly higher for the far-side location than both the middle location, *b* = 0.06, *t*(19) = 15.88, *p* < .001 and the near-side location *b* = 0.03, *t*(19) = 8.48, *p* < .001. Additionally, unlike the raw data, Distance_(S-WG)_ was significantly higher for the near-side location than for the middle location, *b* = -0.03, *t*(19) = -7.39, *p* < .001. Thus, the simulated DRL agent data was found to exhibit the most variability for far-side routes, which aligns with the results for the raw data. However, the simulated DRL agent data exhibited more variability for near-side routes than middle routes, while the far effect was seen in the raw data.

There was also a significant simple main effect of location for humans, *χ*^2^(2) = 1986.00, *p* < .001. Follow-up Bonferroni-corrected pairwise comparisons of Distance_(S-WG)_ for each of the locations revealed that for humans, Distance_(S-WG)_ was significantly higher for the far-side location than both the middle location *b* = 0.44, *t*(191) = 41.22, *p* < .001 and near-side location *b* = 0.37, *t*(191) = 35.28, *p* < .001. Additionally, Distance_(S-WG)_ was significantly lower for the middle location than for the near-side location, *b* = -0.06, *t*(191) = -5.94, *p* < .001. Again, the simulated human data exhibited the most variability for far-side routes which aligns with the results for the raw human data. Further, the simulated human data also exhibited more variability for the near-side than the middle routes, which was also seen in the raw human data.

Collectively, these results indicate that the model was not only able to effectively capture the raw human and DRL agent behavior, but also the differences between the movement trajectories of different human agents and between human and DRL agents. Overall, the simulated human data displayed higher variability (i.e., higher Distance_(S-WG)_) than the simulated DRL agent data across all three route types, and the simulated data for both humans and DRL agents exhibited the most variability in the far-side routes, which is consistent with the results for the raw data. Further, the simulated human data exhibited more variability in the near-side than the middle routes, which was also seen in the raw human data. However, unlike the raw DRL agent data, the simulated DRL agent data was found to exhibit more variability in the near-side routes than the middle routes.

# IN-LAB PARTICIPANTS

A set of twenty in-person participants were recruited to participate in-person on Macquarie University campus for course credit or monetary compensation in order to validate the data collected remotely via Amazon Mechanical Turk (MTurk). Each of the in-person participants performed the task twice, once using a keyboard and once using a joystick (with the ordering counterbalanced). The joystick data was collected in order to compare the trajectories resulting from a continuous controller (joystick) to a discrete controller (keyboard). The joystick controls were as follows: shifting the joystick to the left or right moved the player in that direction and holding down the joystick’s trigger moved the player forward. Other than performing the task using a joystick controller, all other methodology/procedures were the same as for the participants collected via MTurk.

## Confidence Interval Analysis

The confidence interval (CI) analysis was utilized to help determine the similarity between the human trajectories collected via Amazon Mechanical Turk (MTurk) and the human trajectories collected in the lab. For each scenario, the proportion of the mean MTurk trajectory that fell within the 95% CI of the in-lab human trajectories was calculated. This was done separately for the joystick-control and keyboard-control data. The analysis revealed that 100% of the mean MTurk trajectory fell within both the keyboard-controlled and joystick-controlled human 95% CI for all scenarios. Thus, we proceeded with the MTurk data for all analyses in the paper. See Fig. S3 and S4 for example scenarios displaying the mean trajectories and confidence intervals.


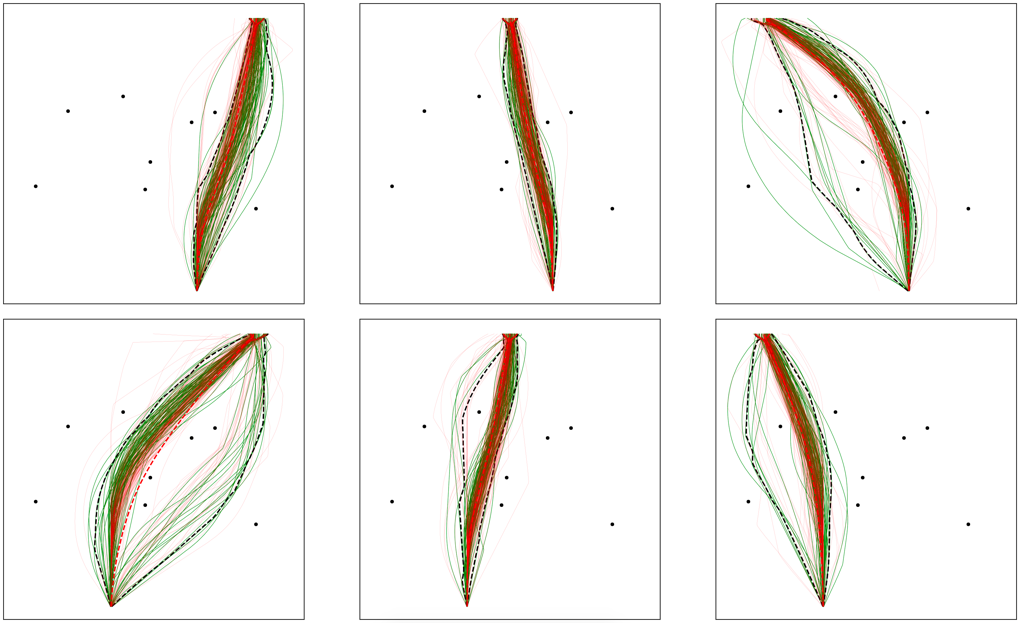

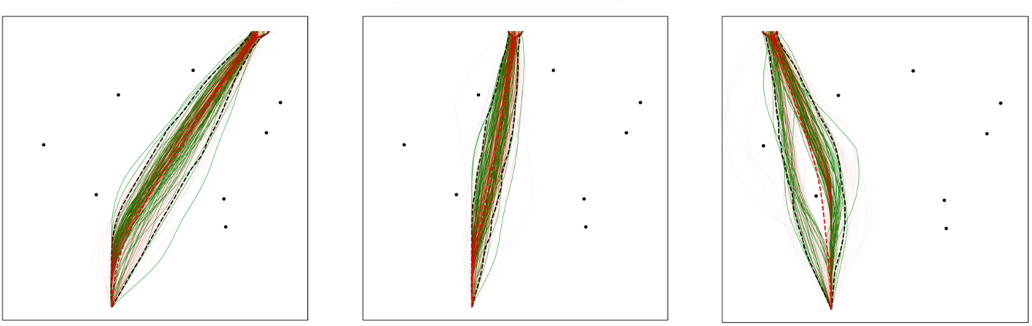


**Figure S3.** Example scenarios with in-lab participant data using keyboard controls in green, and MTurk participant data in red. Mean trajectory for MTurk data is in red and CI for in-lab data in black.


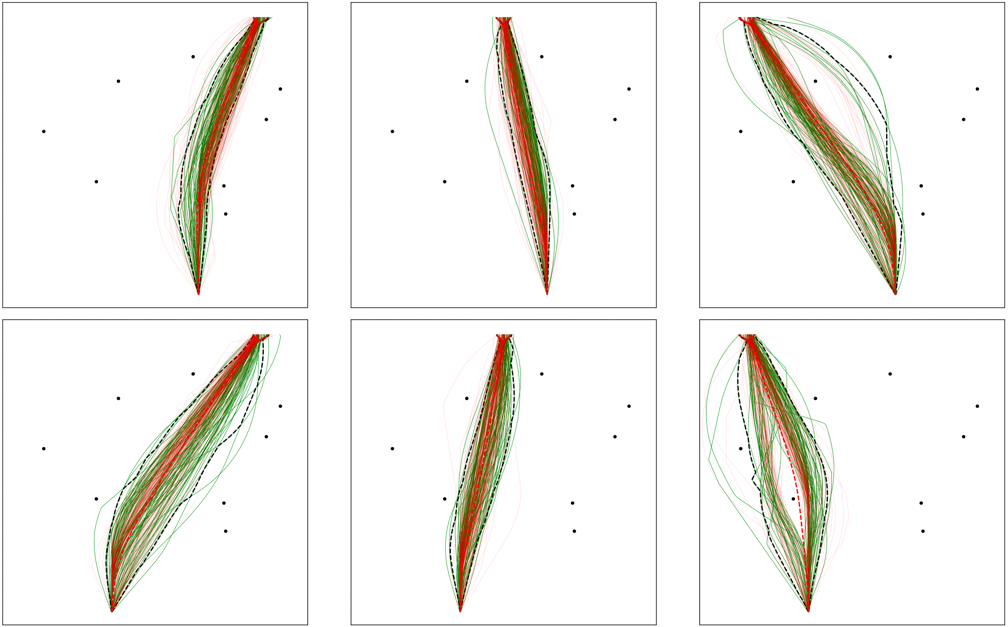

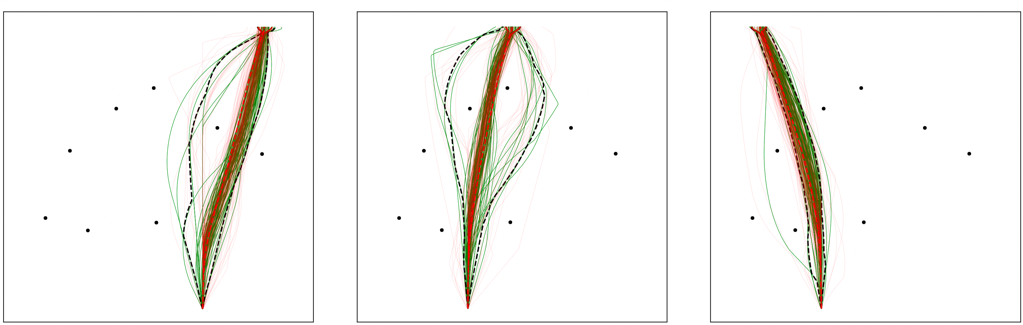


**Figure S4.** Example scenarios with in-lab participant data using joystick controls in green, and MTurk participant data in red. Mean trajectory for MTurk data is in red and CI for in-lab data in black.

# VISUAL DRL AGENT

## Confidence Interval Analysis

The confidence interval (CI) analysis was utilized to help determine the similarity between the raw human and visual DRL agent trajectories. For each scenario, the proportion of the mean visual DRL agent trajectory that fell within the 95% CI of the human trajectories was calculated. The analysis revealed that 97.78% of the mean visual DRL agent trajectory fell within the human trajectories, with a standard deviation of 11.76. See Figure S5 for example scenarios displaying the visual DRL agent trajectories.


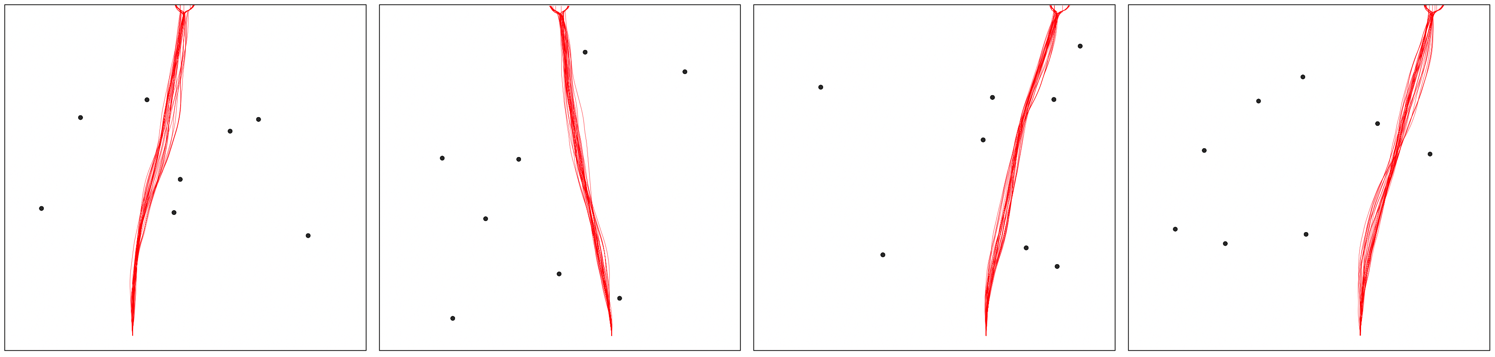


**Figure S5.** Example scenarios with visual DRL agent trajectories in red.

# RAYCAST DRL AGENT TRAINED WITHOUT TARGET INFORMATION

## Confidence Interval Analysis

The confidence interval (CI) analysis was utilized to help determine the similarity between the raw human trajectories and the trajectories from the raycast DRL agents trained without target information (Raycast_NT_). For each scenario, the proportion of the mean Raycast_NT_ trajectory that fell within the 95% CI of the human trajectories was calculated.

The analysis revealed that ﻿91.67% of the mean Raycast_NT_ trajectory fell within the human trajectories, with a standard deviation of 17.55. See Figure 6 for example scenarios displaying the Raycast_NT_ trajectories.


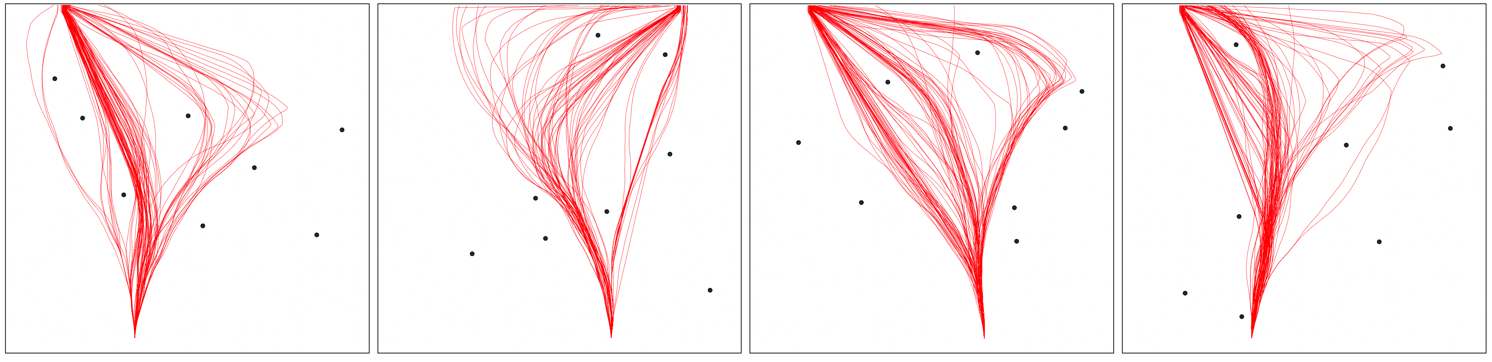


**Figure S6***.* Example scenarios with Raycast_NT_ trajectories in red.

# OBSTACLE SCENARIOS

With (0,0) at the center of the playing area (refer to Figure 2 in the main paper), the two start positions (x,y) and three target positions (x,y) were (-5,-16) and (5,-16) and (-10,16), (0,16), and (10,16), respectively. The obstacle positions for the 18 obstacle configurations are listed in the table below.

| Obstacle Config  No. | Obstacle | | | | | | | | | | | | | | | |
| --- | --- | --- | --- | --- | --- | --- | --- | --- | --- | --- | --- | --- | --- | --- | --- | --- |
|  | 1 | | 2 | | 3 | | 4 | | 5 | | 6 | | 7 | | 8 | |
|  | X | Y | X | Y | X | Y | X | Y | X | Y | X | Y | X | Y | X | Y |
| 1 | 7.1 | 4.8 | 11.9 | -6.4 | -13.8 | -3.8 | 4.4 | 3.7 | -0.4 | -1.0 | -1.0 | -4.1 | -3.6 | 6.7 | -10.0 | 5.0 |
| 2 | -12.7 | 8.8 | 12.4 | -6.2 | 6.5 | 0.3 | -6.1 | -2.3 | 14.9 | 3.9 | 0.1 | 5.2 | 1.5 | -5.3 | -10.0 | 5.0 |
| 3 | -8.0 | -7.1 | 11.7 | 1.5 | -4.8 | 6.6 | -12.8 | -5.8 | 6.7 | 4.4 | -0.2 | -6.2 | -0.5 | 8.9 | -10.0 | 1.8 |
| 4 | 14.4 | 7.6 | -4.2 | 8.5 | 8.1 | -6.7 | 7.9 | -3.5 | 4.3 | 11.3 | -6.8 | -3.0 | 12.8 | 4.1 | -12.8 | 2.7 |
| 5 | 14.5 | -11.4 | 10.2 | 11.1 | 4.6 | -3.9 | 3.7 | 13.0 | -1.3 | -6.5 | -8.4 | -8.0 | -2.3 | -2.6 | 10.6 | 1.6 |
| 6 | 4.7 | 2.9 | -4.9 | -8.2 | 8.9 | -7.5 | -10.9 | 8.0 | 5.6 | 7.0 | 11.8 | -9.3 | 11.5 | 6.8 | 14.0 | 11.9 |
| 7 | -0.7 | -6.5 | 14.3 | 1.5 | -12.8 | 1.3 | -5.3 | -8.8 | 1.0 | 0.1 | 5.5 | 3.3 | 4.4 | -7.2 | 4.9 | -7.8 |
| 8 | 12.6 | 5.4 | 5.2 | 12.0 | 12.2 | -13.5 | 5.0 | -11.2 | -0.4 | -8.0 | 1.7 | 1.4 | -6.2 | 12.8 | -4.1 | -12.3 |
| 9 | -9.6 | 9.8 | 8.8 | -13.7 | -3.2 | -7.7 | -7.7 | 4.0 | -8.0 | -2.1 | -13.7 | -3.7 | 7.8 | 1.6 | -2.2 | 9.8 |
| 10 | 1.9 | 6.2 | 9.2 | 5.3 | 11.1 | -10.4 | -4.0 | -0.1 | -0.8 | -1.8 | 12.7 | 5.0 | -10.7 | 1.6 | 9.5 | -2.2 |
| 11 | -7.2 | -4.7 | -4.0 | 1.0 | 5.7 | -12.4 | 12.0 | 9.5 | -11.4 | 1.1 | 2.4 | 11.4 | -10.3 | -14.3 | -0.1 | -10.0 |
| 12 | -6.5 | 12.1 | -6.0 | -14.0 | -6.2 | -4.4 | 7.2 | -6.8 | 14.0 | 4.1 | 13.3 | 10.0 | -11.4 | -11.7 | 4.1 | 2.5 |
| 13 | 6.7 | 3.7 | -0.1 | 10.4 | 0.8 | 9.5 | -9.1 | -11.6 | 2.5 | -0.1 | -12.7 | 6.1 | -10.6 | -10.9 | -6.0 | -1.9 |
| 14 | 2.9 | -3.8 | -6.4 | -5.6 | -4.4 | 7.7 | -2.6 | -4.7 | -14.0 | -13.1 | 0.9 | 11.9 | 1.4 | -9.3 | 1.9 | -7.0 |
| 15 | 0.9 | 5.4 | -2.7 | 11.3 | 14.1 | 1.6 | 7.3 | 10.8 | 7.0 | -6.7 | -5.6 | -10.6 | 4.8 | 2.3 | -13.9 | -5.3 |
| 16 | 14.0 | 7.2 | 8.0 | 3.4 | -11.0 | 13.5 | -10.7 | 5.8 | -0.3 | 4.1 | -4.2 | 1.8 | -1.3 | -11.1 | -5.2 | 14.6 |
| 17 | -7.0 | 8.0 | -14.4 | -9.6 | -12.9 | -11.7 | 8.5 | 8.9 | -11.6 | 1.6 | -0.1 | 11.8 | 2.6 | 8.8 | -14.0 | -8.6 |
| 18 | 2.4 | 5.7 | -14.6 | -13.0 | -7.0 | -8.5 | 0.6 | 12.8 | -7.8 | 10.7 | 0.3 | -0.6 | 7.3 | -1.8 | -14.0 | 8.7 |

# ASSESSMENT OF EVOLUTION OF TRAJECTORY VARIABILITY

A repeated measures ANOVA was utilized to examine the cumulative distance of the trajectories (i.e., path length) for each participant over the course of each third of the trials (i.e., early, middle, and end trials) within each scenario, to determine whether variability was significantly different across the different stages of the experiment. The analysis revealed no significant effect of stage for humans, *F*(2, 352) = 1.08, *p* = .34, *η_p_²* = .006, or for the DRL agents, *F*(1, 19) = .04, *p* = .85, *η_p_²* = .002, indicating that variability did not differ across repeated trials. The lack of apparent learning or tuning observed for the human participants is likely due to the simplicity of the task utilized.
